# Competencies for medical nutritional counselling of children and adolescents: Analysis of NKLM 2.0 based on an evidence-based catalogue of criteria

**DOI:** 10.3205/zma001854

**Published:** 2026-06-15

**Authors:** Lena Sophie Rudolf, Cathleen Bunzel, Lisa-Michelle Dietz, Katja Kröller, Jana Markert, Helen Clara Schörghofer, Mario Meixner, Laura von Iven, Anke Lux, Anke Rissmann

**Affiliations:** 1 Otto von Guericke University Magdeburg, Medical Faculty, Malformation Monitoring Centre Saxony-Anhalt, Magdeburg, Germany; 2 Anhalt University of Applied Sciences, Department of Agriculture, Ecotrophology and Landscape Development, Köthen, Germany; 3 Dresden University of Technology, Institute for Vocational Education and Vocational Didactics, Dresden, Germany; 4 University Hospital Cologne Centre for Integrated Oncology CIO Cologne, Internal Medicine I, Cologne, Germany; 5 Otto-von-Guericke-University Magdeburg, Medical Faculty, Institute of Medical Data Science, Magdeburg, Germany

**Keywords:** qualitative research, undergraduate medical education, competency-based education, National Competence-Based Catalogue of Learning Objectives (NKLM), curriculum analysis, medical nutrition education

## Abstract

**Objective:**

Breastfeeding and Nutrition Counselling (NC) plays an important role in paediatric and adolescent medicine for the prevention of nutrition-related diseases. The necessary fundamentals should already be taught during undergraduate medical education.

The objectives of this paper were 1. to analyse the National Competence-Based Catalogue of Learning Objectives 2.0 (NKLM) with regard to nutrition-related competencies, 2. to identify possible discrepancies, and 3. to formulate recommendations.

**Methodology:**

With the help of a structured literature research, the competency goals which are necessary for primary preventive NC were identified and summarised in a theoretical catalogue of criteria. The NKLM 2.0 was subsequently checked for its content using the qualitative content analysis according to Mayring.

**Results:**

The final code system consisted of 82 codes. All 1,426 coded segments were distributed across the code categories as follows: “Preventive nutritional knowledge”: 532 (37.31%), “Communication competencies”: 442 (31%), “NC competencies”: 216 (15.15%), “Nutritional medicine”: 236 (16.55%). No teaching content was identified for 5 codes. Teaching formats and time frames were not specified.

**Conclusion:**

NKLM 2.0 covers most of the competencies required for adequate preventive medical NC. There are gaps in nutritional knowledge (nutrition of breastfeeding mothers, cooking and kitchen techniques, eating behaviour) and in nutrition counselling competencies (nudging, sensitive communication about body weight, systemic counselling). These topics can be taught in a targeted manner in communication courses or as part of culinary medicine programs. Mandatory advanced training in paediatrics and adolescent medicine, as well as clear guidelines regarding teaching formats and time requirements, appear to be sensible measures to ensure lasting learning success and effective prevention.

## Zusammenfassung

**Zielsetzung:**

Für die Prävention ernährungsassoziierter Erkrankungen spielen Still- und Ernährungsberatungen (EB) in der Kinder- und Jugendmedizin eine wichtige Rolle. Dafür benötigte Grundlagen sollten bereits im Medizinstudium vermittelt werden.

Ziele dieser Arbeit waren 1. Analyse des Nationalen Kompetenzbasierten Lernzielkatalogs Medizin 2.0 (NKLM) bezüglich ernährungsbezogener Kompetenzen, 2. Aufdecken möglicher Diskrepanzen und 3. Formulieren von Empfehlungen.

**Methodik:**

Mithilfe einer strukturierten Literaturrecherche wurden die für eine primärpräventive EB notwendigen Kompetenzziele ermittelt und in einem theoretischen Kriterienkatalog zusammengefasst. Der NKLM 2.0 wurde anschließend mittels qualitativer Inhaltsanalyse nach Mayring auf deren Beinhaltung geprüft.

**Ergebnisse:**

Das finale Codesystem umfasste 82 Codes. Alle 1,426 codierten Segmente verteilten sich wie folgt auf die Codekategorien: „Ernährungswissen präventiv“: 532 (37,31%), „Kompetenzen Kommunikation“: 442 (31%), „Kompetenzen EB“: 216 (15,15%), „Ernährungsmedizin“: 236 (16,55%). Zu 5 Codes wurden keine Lehrinhalte identifiziert. Lehrformate und Zeitumfänge waren nicht angegeben.

**Schlussfolgerung:**

Der NKLM 2.0 umfasst den Großteil der für eine adäquate ärztliche präventive EB erforderlichen Kompetenzen. Defizite bestehen im Ernährungswissen (Ernährung der Mutter in der Stillzeit, Koch- und Küchentechniken, Essverhalten) sowie in Beratungsstrategien (Nudging, sensibler Umgang mit Körpergewicht, systemische Beratung). Diese Inhalte können gezielt in Gesprächsführungskursen oder im Rahmen von Culinary-Medicine-Angeboten vermittelt werden. Eine verpflichtende Vertiefung in der Fachärzt:innenweiterbildung Kinder- und Jugendmedizin sowie klare Vorgaben zu Lehrformaten und Zeitumfängen erscheinen zur Sicherung nachhaltiger Lernerfolge und effektiven Prävention sinnvoll.

## 1. Introduction

### 1.1. Overview

Nutrition-related diseases belong to the most common preventable health problems and cause significant economic costs [1], [2], [3].

Given that one in six deaths in Europe is attributable to unfavourable dietary habits, a preventive diet is important in order to reduce the long-term burden of disease and mortality among patients [2].

Even in the first years of life, breastfeeding and nutritional education can have preventive effects and sustainably reduce the risk of developing excess weight [4], [5], [6], [7]. 

Childhood is a sensitive phase for the development of eating habits, which often remain in place throughout life and have a significant impact on the risk of nutrition-related diseases [8], [9], [10]. 

In this context, medical nutrition counselling (NC) represents a key approach to behavioural prevention and can be implemented effectively, particularly in the context of early detection examinations by paediatricians [8], [11].

Numerous studies have shown that doctors and medical students consider being competent in NC to be part of their professional responsibility, but rate their own counselling competencies and the curricular integration of nutrition-related content as insufficient [12], [13], [14], [15], [16], [17]. 

### 1.2. National Competence-Based Catalogue of Learning Objectives (NKLM)

Deficits in medical nutrition education have been known for decades [18]. Efforts are being made worldwide to reach a consensus on content and curricular integration [19], [20], [21], [22], [23], [24]. Nevertheless, nutrition education remains insufficiently anchored in medical training; for example, up to 75% of faculties in the United Kingdom and the United States of America do not offer mandatory courses about clinical nutrition [25], [26], [27].

In Germany, medical faculties are guided by the Licensing Regulations for Physicians (ÄApprO) [https://www.gesetze-im-internet.de/_appro_2002/BJNR240500002.html], which have traditionally focused on imparting factual knowledge. The “German Master Plan for medical education 2020” has initiated a paradigm shift: in the future, medical competencies, communication and practical skills are to be taught on an equivalent basis with theoretical knowledge [28]. 

**Figure 1:**
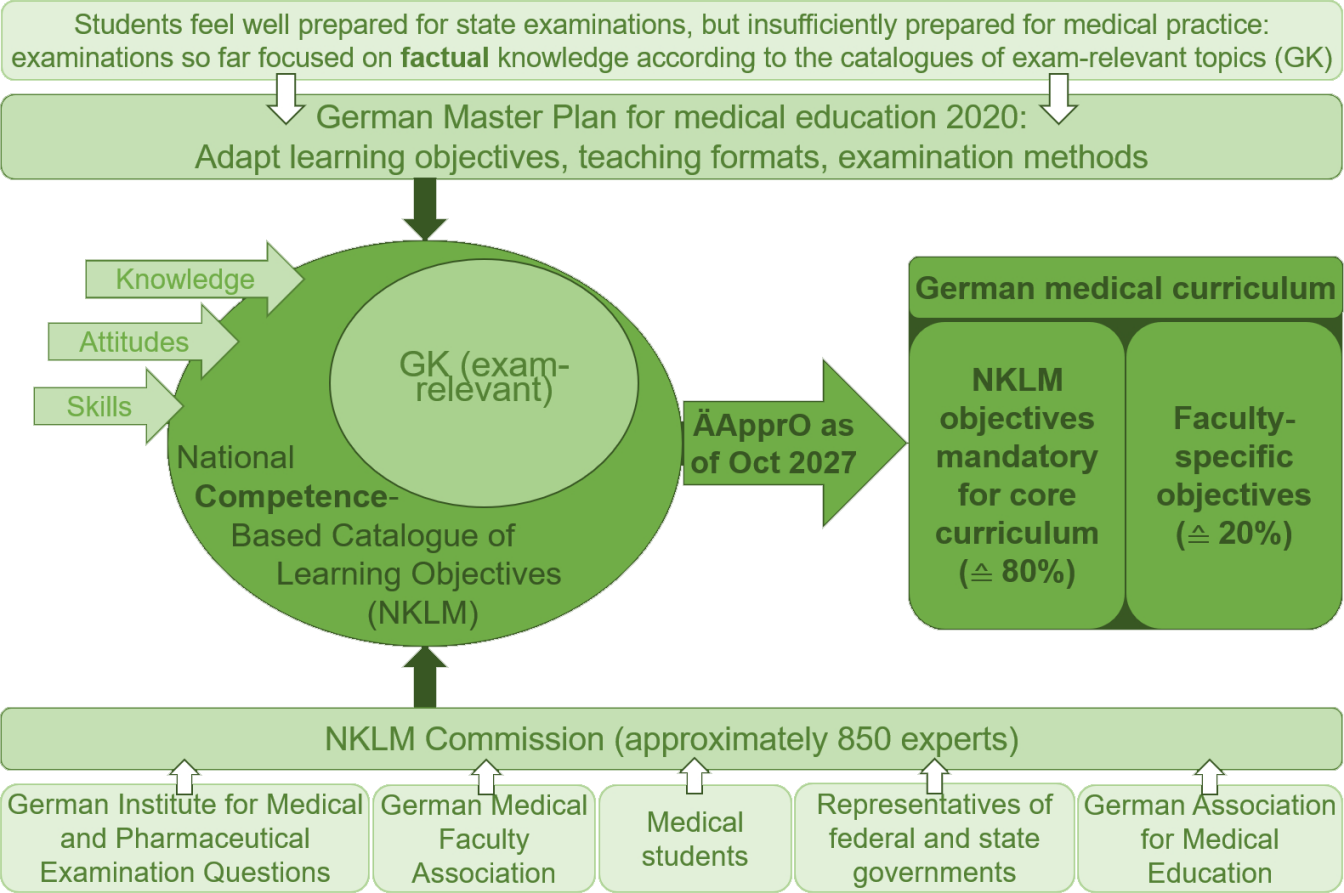
Illustration of relations between catalogues of exam-relevant topics (GK), National Competence-Based Catalogue of Learning Objectives (NKLM) and the prospective medical curriculum in Germany Own figure, content from: [https://www.impp.de/über-uns/entwicklung-des-impps/aufgaben.html] last checked on 01/16/2025, [https://www.impp.de/pruefungen/allgemein/gegenstandskataloge.html], last checked on 01/16/2025, [29], [30], [31]. ÄApprO: Licensing Regulations for Physicians

As part of this process, the National Competence-Based Catalogue of Learning Objectives (NKLM) was developed (currently version 2.0) [29]. With the new ÄApprO, which is expected to come into force on 1 October 2027, 80% of the curriculum of medical studies will in future be required to consist of content from the NKLM 3.0, which will have been further developed by then [30], [31], [https://nklm.de/zend/menu] (see figure 1 (Fig. 1)).

### 1.3. Objective

In the first step, the required competencies for adequate preventive NC by paediatricians and health care providers were identified. These were recorded in a theoretical catalogue of criteria (target state).

On this basis, NKLM 2.0 was analysed in terms of nutritional knowledge, communication and counselling competencies (current state). In addition, existing teaching formats and time frames were recorded. The aim was to compare target and actual status in order to identify gaps and formulate specific recommendations.

## 2. Methods

### 2.1. Development of the theoretical basis

From March to June 2023, structured literature research was conducted with the help of PubMed, Scopus and publications from professional associations to identify key content, areas of expertise and other influencing factors of an adequate primary preventive NC. Only aspects that were confirmed by at least two peer-reviewed publications were included in the theoretically derived catalogue of criteria. The detailed research strategy is presented in attachment 1 . The catalogue of criteria initially comprised five main categories with associated meta and learning objectives, which can be found in attachment 2 . In the course of the curriculum analysis, the code category “nutritional medicine” was added inductively. A total of four categories were used for evaluation: “preventive nutritional knowledge”, “communication competencies”, “NC competencies” and “nutritional medicine”.

### 2.2. Qualitative content analysis

NKLM 2.0 is available as an online version [https://nklm.de/zend/menu]. For the analysis, a 1,467-page PDF version was generated on 24 July 2023. The qualitative content analysis was performed based on Mayring [32] and with the help of the MAXQDA Analytics Pro software (version 2022).

The categories of the theoretical catalogue of criteria were first converted into a deductive code system, whereby thematically consistent criteria (up to five) were grouped together to individual codes. Code memos were formulated for each code (see attachment 2 ).

Regular consultations took place during the coding process between the three coders, who applied their coding system within the framework of the superordinated research project. On this basis, codes and code memos were gradually refined and an intersubjective consensus was ensured. 

The coding of NKLM 2.0 was carried out by one person.

In addition, inductive codes were developed to map further curricular content. Inductive, primary preventive codes were assigned to the categories 1 to 3; the inductive category “6. Nutritional medicine” was used to record nutritional medical content. The final code system consisted of 43 deductive and 39 inductive codes. Additionally, teaching formats and time frames were extracted.

### 2.3. Statistical analysis

The tables and graphs were created by using Microsoft 365. Data analysis was performed by using IBM SPSS Statistics (version 29). The distribution of coded segments across the code categories was tested by using the chi-square goodness-of-fit test.

## 3. Results

**Figure 2:**
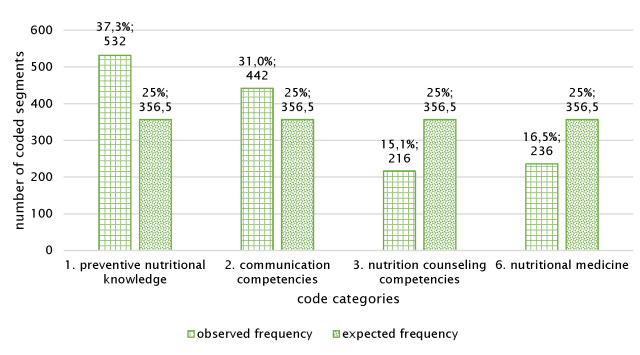
Distribution of coded segments in relation to nutrition counselling competencies in the National Competence-Based Catalogue of Learning Objectives 2.0 across the code categories. Chi-square goodness-of-fit test: The observed code frequencies are not normally distributed (p<0.001)

A total of 1,426 text segments were coded. Figure 2 (Fig. 2) shows their distribution across the code categories.

### 3.1. Code category “1. Preventive nutritional knowledge”

Table 1 (Tab. 1) shows the number of segments coded per code in the first code category. The 10 rules of the German Nutrition Society, the importance of a balanced, predominantly plant-based diet and the influence of highly processed foods were documented in the codes with more general content. A prevention of nutrition-related diseases, children's food preferences and aversions as well as healthy eating in schools and day care centres were also mentioned. Several topics relating to the connection between nutrition and the microbiome and the anatomy and physiology of the digestive tract were identified.

With regard to nutritional assessment, anthropometric data such as body weight, body mass index and percentiles were frequently coded to assess nutritional status. The term nutritional history was also coded. 

In the coded segments about interdisciplinary care, its necessity was mentioned in the context of NC and lifestyle changes. Dietitiants were explicitly mentioned as contact persons.

In the coded segments about breastfeeding, its advantages and disadvantages, physiology, management as well as psychological and social factors were discussed. Learning objectives such as “being able to describe different types of breastfeeding difficulties”, “avoiding milk engorgement” and appropriate treatment measures for mastitis were also mentioned. Drinking behaviour and problems of infants as well as formula feeding were also discussed.

Learning content of NKLM 2.0 about eating behaviour covered topics such as polyphagia and binge eating, feeding disorders in infancy and early childhood, and eating as psychosocial compensation, particularly in paediatric and adolescent medicine. In addition, skills relating to knowledge and questioning of risks and early warning signs for eating disorders were listed in the context of primary prevention.

Altogether, almost all knowledge competencies required for primary preventive NC are covered in NKLM 2.0. The only exception is specific content about cooking and kitchen techniques and about nutrition for mothers during breastfeeding.

### 3.2. Code category “2. Communication competencies”

Table 2 (Tab. 2) shows the number of coded segments within the second code category. Most segments were assigned to the higher-level communication code. Topics such as conversation techniques, the importance of communication and empathetic interaction with patients were frequently identified. Specific terms such as “medical”, “patient-centred” or “doctor-patient communication”, “relationship building” as well as references to “shared decision making” were also mentioned. In addition, the explicit mention of patient- or family-centred care was documented.

The topic “dealing with difficult situations” included content about communication in crisis and conflict situations, de-escalation strategies, and communication with people with perception or communication disorders. Another learning objective mentioned was the ability to communicate appropriately with “difficult” patients, including children and adolescents with behavioural problems.

The segments about intercultural competencies mainly related to dealing with language barriers and adapting medical communication to the socio-cultural background of patients.

Under the code of communication in paediatrics, content relating to conversations with parents, relatives, children and adolescents was coded. Explicit reference was made to adapting language to the respective cognitive stage of development.

In conclusion, all communication competencies were demonstrated multiple times in NKLM 2.0.

### 3.3. Code category “3. Nutrition counselling competencies”

Table 3 (Tab. 3) shows the number of coded segments within the third code category. The majority of segments fell under the more general codes and mainly included phrases such as “perform NC”, “advise about healthy eating” or “conduct counselling sessions”. In addition, references to the transtheoretical model of behaviour change, to motivational interviewing and resource- and solution-oriented communication were identified.

Several segments addressed physicians’ role modeling, self-care, and self-efficacy expectations, but without a specific reference to nutrition.

The most frequent inductive code referred to the identification and integration of contextual factors according to the biopsychosocial model.

Overall, almost all counselling competencies required for primary preventive NC are covered by NKLM 2.0. Content on nudging or sensitive communication about body weight could not be found.

### 3.4. Code category “6. Nutritional medicine”

Table 4 (Tab. 4) shows the number of segments coded per code in the sixth code category.

Here, most segments were classified under “6.1.3.1. Cachexia, sarcopenia, malnutrition, undernourishment” and “6.9.4. Eating disorders”. The terms malnutrition, undernourishment or cachexia were primarily used here without further details. With regard to eating disorders, the learning objectives focused in particular on the diagnosis and treatment of eating disorders, including diagnostic criteria, potentially abnormal laboratory parameters, comorbidities and the conditions for initiating compulsory measures. Furthermore, content relating to adverse outcomes (electrolyte imbalances, electrocardiogram changes, oesophageal rupture, refeeding syndrome) could be coded.

In NKLM 2.0, the roles of highly processed foods and a predominantly animal-based diet were mentioned in relation to nutritional medicine and obesity. Another point was a fundamental change of eating habits that is often necessary for weight loss in children.

### 3.5. Time frames and teaching formats

NKLM 2.0 did not specify time frames, but rather recommendations for the semester or academic year and the level of competence to be achieved regarding learning objectives. 

Teaching formats were not included, and recommendations were rarely mentioned, for example, “Can be implemented very well in communication courses”.

## 4. Discussion

### 4.1. Results of the analysis

The NKLM 2.0 contained all except four of the theoretically derived learning objectives. The topics of maternal nutrition during breastfeeding, cooking and kitchen techniques and the nutrition of infants from the field of preventive nutritional knowledge were not covered. Furthermore, nudging and sensitive handling of body weight from the NC category were missing. In addition, numerous inductively recorded contents were identified, most of which had a focus on nutritional medicine.

A working group of the European Society for Clinical Nutrition and Metabolism (ESPEN) identified nutrition-related learning objectives that medical students should have mastered by the end of their studies [33], [34] as part of the “Nutrition Education in Medical Schools” project. All of this content was found both in the codes of the code system which was used in this study and in the NKLM 2.0. However, it was noticeable that the proposal by ESPEN lacked content about communication and NC as defined in the code system used in this study. It only included aspects of nutritional science and nutritional medicine [34].

Against the background of the ongoing development and reduction of the NKLM, it is possible that not all identified content will be included in the final version [35]. However, advisory skills should not be reduced but rather expanded. Motivational interviewing in particular is a key tool for empowering patients to adopt healthy eating habits [36]. Counselling competencies also play an important role in further areas with preventive potential, such as addiction therapy or promotion of regular physical activity [37], [38].

Following the entry into force of the new ÄApprO, NKLM 3.0 will become the binding core curriculum for medical faculties in Germany [30], [31]. Such a core curriculum will enable comprehensive and nationally standardised medical training, as it is an aim in the future and already partly implemented internationally [39]. It also specifies precisely which skills students should have acquired after completing a single teaching unit and which competencies graduates should have acquired after completing the entire course of study. This creates uniform conditions for subsequent specialist medical training and helps to ensure a high quality of care [40]. The NKLM thus represents a key opportunity to improve nutritional education at medical schools.

#### 4.1.1. Breastfeeding

An impressive example of the long-term impact of infant nutrition is the protective effect of breastfeeding in relation to the development of obesity, allergies and acute and chronic diseases [41], [42], [43]. 

In a meta-analysis, Kehinde et al. (2023) were able to show in the 14 included studies (2014-2021) that the most effective interventions of successful breastfeeding are evidence-based education and breastfeeding support [44]. The importance of this topic has already been addressed in the National Strategy for Breastfeeding Promotion (2021), which calls for the integration of breastfeeding-related content into education, training and continuing education programs for relevant medical professions [5]. The planned S3 guideline “Breastfeeding duration and interventions to promote breastfeeding” is expected to serve as a scientific foundation for the future development of breastfeeding-related competencies in the NKLM [45].

The analysed NKLM 2.0 contained key information about breastfeeding that all medical graduates should be able to apply. However, given the important role that paediatricians play in breastfeeding counselling, it seems necessary to significantly deepen knowledge and counselling competencies about breastfeeding in subsequent specialist training [46].

#### 4.1.2. Time frames and teaching formats

As a result of the decisions made by the “Master Plan for medical education 2020,” competency-based medical training formats should become an integral part of medical studies, while new examination formats should replace the current multiple-choice question format [28], [29]. The analysis result that no teaching formats were specified in NKLM 2.0 was therefore unexpected, especially since these play an important role in the learning success of medical students, whereby practice-oriented methods achieve the best results [47], [48].

In recent years, various medical schools around the world have established courses in so-called culinary medicine, which offer medical students the opportunity to acquire practical skills in the field of NC [49], [50], [51]. 

This is a forward-looking approach, as nutritional knowledge acquired through conventional teaching methods (lectures, seminars) can only be applied to a limited extent in practice [20], [52]. The association Culinary Medicine Deutschland e.V. collaborated with the University Medical Centre Göttingen to develop the first German pilot project which is called "culinary medicine" and mainly takes place in a teaching kitchen [21].

Participation in this elective course led to a significant improvement of the students' counselling competencies, nutritional knowledge, attitude towards NC in medical practice, well-being and eating habits [21]. This format makes it possible to learn cooking and kitchen techniques that are not covered by NKLM 2.0. Since medical studies involve teaching a great amount of content, it seems difficult to implement mandatory culinary medicine courses at this point of time [53], [54].

Methods that have already been implemented in medical schools include problem-oriented learning, which has proven to be an effective method for medical students to acquire new skills [55]. In addition, communication competencies can be taught in a targeted and sustainable manner in conversation courses, as an example from Charité Berlin shows. Here, a communication curriculum was successfully integrated into the superordinated curriculum [56]. Building on this, the authors recommend to integrate nutrition-related competencies both horizontally and vertically and to teach them in practical teaching formats from the start of studies through to specialist medical training [53], [57], [58] .

### 4.2. Method discussion

Kondracki et al. (2002) described qualitative content analysis as a method for researching nutrition education in the health sector [59]. In addition, Amini-Rarani et al. (2021) and Malekmohammadi et al. (2023) successfully conducted qualitative content analyses using MAXQDA regarding medical competencies. As part of a study about the care expectations of rehabilitation patients with migrant background in Germany by Brzoska et al. (2017), the targeted application of qualitative content analysis according to Mayring (including deductive and subsequent inductive code category formation) in combination with the MAXQDA software was extensively presented in interviews and documents [60]. It can be concluded that a suitable methodological approach was used here to identify NC competencies contained in NKLM 2.0.

This study combined qualitative and quantitative methods: in addition to covering the code system by NKLM 2.0, codes that were used particularly frequently or rarely were examined for their wording and detailed learning content. In this way, the frequency of a code or code category could be used to substantiate its qualitative significance, as described by Mayring (2022) [61].

Another strength of the coding process was the manual approach, which means that it can be assumed that all nutrition-related curricular content was captured. Sources of error that exist in dictionary-based term counts which are performed by computer programs [62] could be avoided in this way. The transferability of classic quality criteria onto content analysis research is the subject of critical discussion [62], [63]. Determination of intercoder reliability is frequently performed [64]. This was not possible because NKLM 2.0 was coded by only one person. Due to time restrictions, the alternative reliability check using the intracoder reliability had to be omitted [65]. In future work, a reliability check should be attempted.

## 5. Conclusion

NKLM 2.0 contains most of the learning and competency objectives which are required for adequate medical preventive NC. Gaps are evident in the areas of nutritional knowledge and counselling strategies. It is not clear which of the included competencies will remain in the final version of NKLM 3.0. Paediatricians should acquire additional competencies in breastfeeding counselling, complementary feeding and specific paediatric nutrition topics during their specialist training. The NKLM presents, as the future mandatory core curriculum for medical studies, an important opportunity to improve medical nutrition education for physicians in Germany. Specific details regarding teaching formats and time frames for individual learning objectives would be desirable for optimal learning success.

## Abbreviations

- ÄApprO: German Licensing Regulations for Physicians
- NC: Nutritional counselling
- ESPEN: European Society for Clinical Nutrition and Metabolism
- GK: Catalogues of exam-relevant topics
- NKLM: National Competence-Based Catalogue of Learning Objectives

## Notes

### Availability of data and material

The data sets used and/or analysed in the current study are available upon request from the corresponding author or are included in the supplementary information files (see criteria catalogue as attachment 2 and literature research strategy as attachment 1 ), accompanying this article.

### Funding

This work was funded by the Federal Ministry of Agriculture, Food and Regional Identity (BMLEH) based on a resolution passed by the German Bundestag. The project was managed by the Federal Office for Agriculture and Food (BLE), funding codes 2822HS001, 2822HS005, 2822HS006.

### Authors’ ORCIDs

- Lena Sophie Rudolf: [0009-0009-1689-767X]
- Cathleen Bunzel: [0009-0000-2607-7012]
- Lisa-Michelle Dietz: [0009-0003-7689-8291]
- Katja Kröller: [0000-0003-3016-7453]
- Jana Markert: [0009-0004-1811-1304]
- Helene Clara Schörghofer: [0009-0009-1007-5237]
- Mario Meixner: [0009-0004-8418-2338]
- Laura von Iven: [0009-0000-4994-916X]
- Anke Lux: [0000-0001-5116-7116]
- Anke Rissmann: [0000-0002-9437-2790]

## Competing interests

The authors declare that they have no competing interests. 

## Supplementary Material

Literature search strategy

Theoretical catalogue of criteria with assigned codes and code memos

## Figures and Tables

**Table 1:**
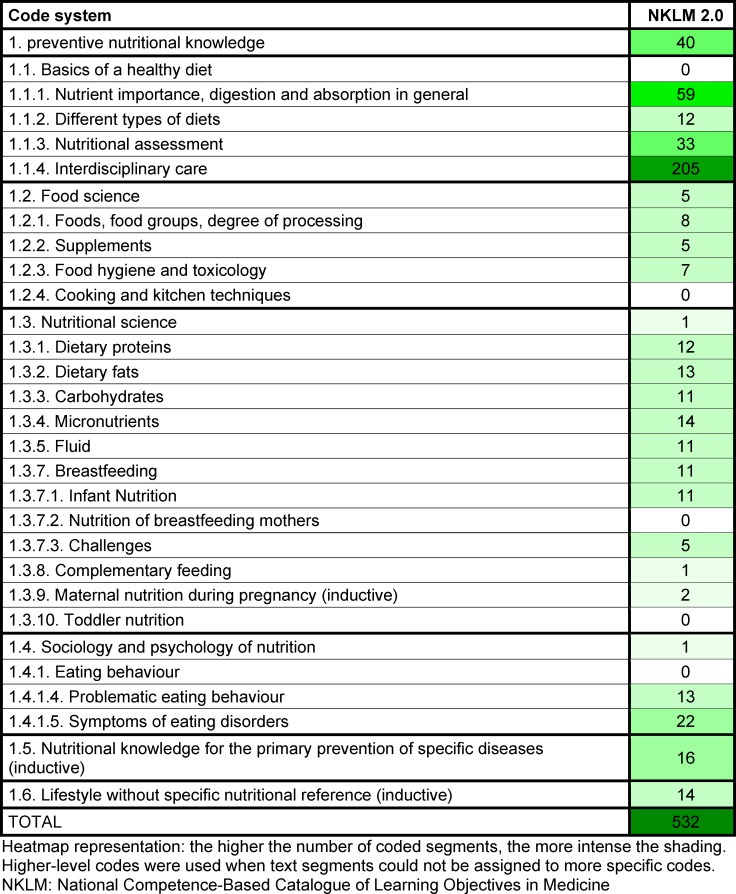
Number of segments coded per code in the NKLM 2.0 in the code category “1. Preventive nutritional knowledge”

**Table 2:**
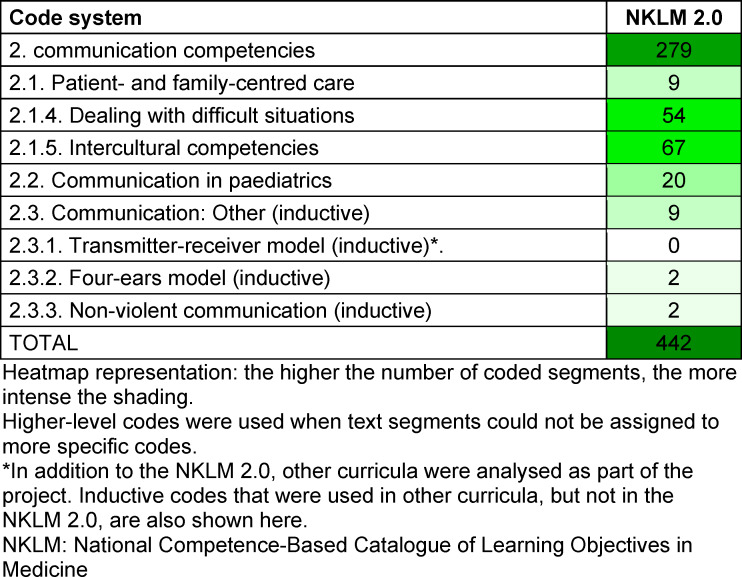
Number of segments coded per code in the NKLM 2.0 in the code category “2. Communication competencies”

**Table 3:**
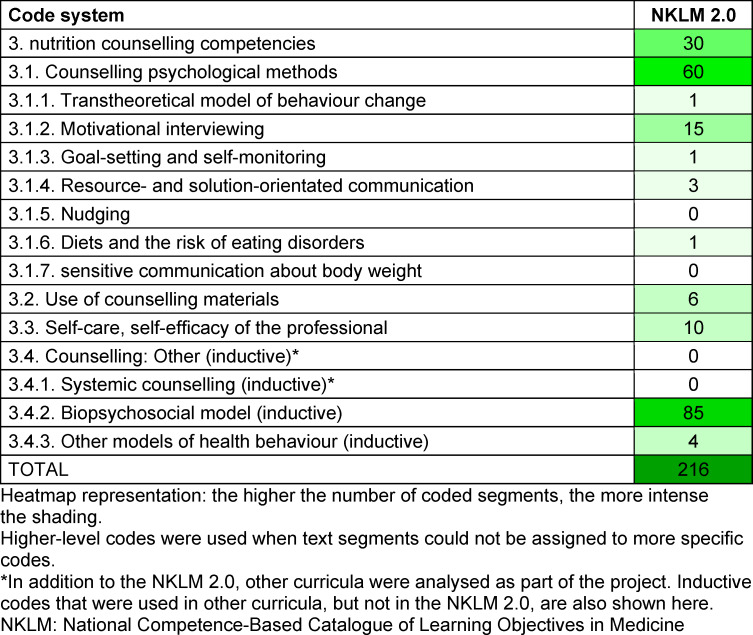
Number of segments coded per code in the NKLM 2.0 in the code category “3. Nutrition counselling competencies”

**Table 4:**
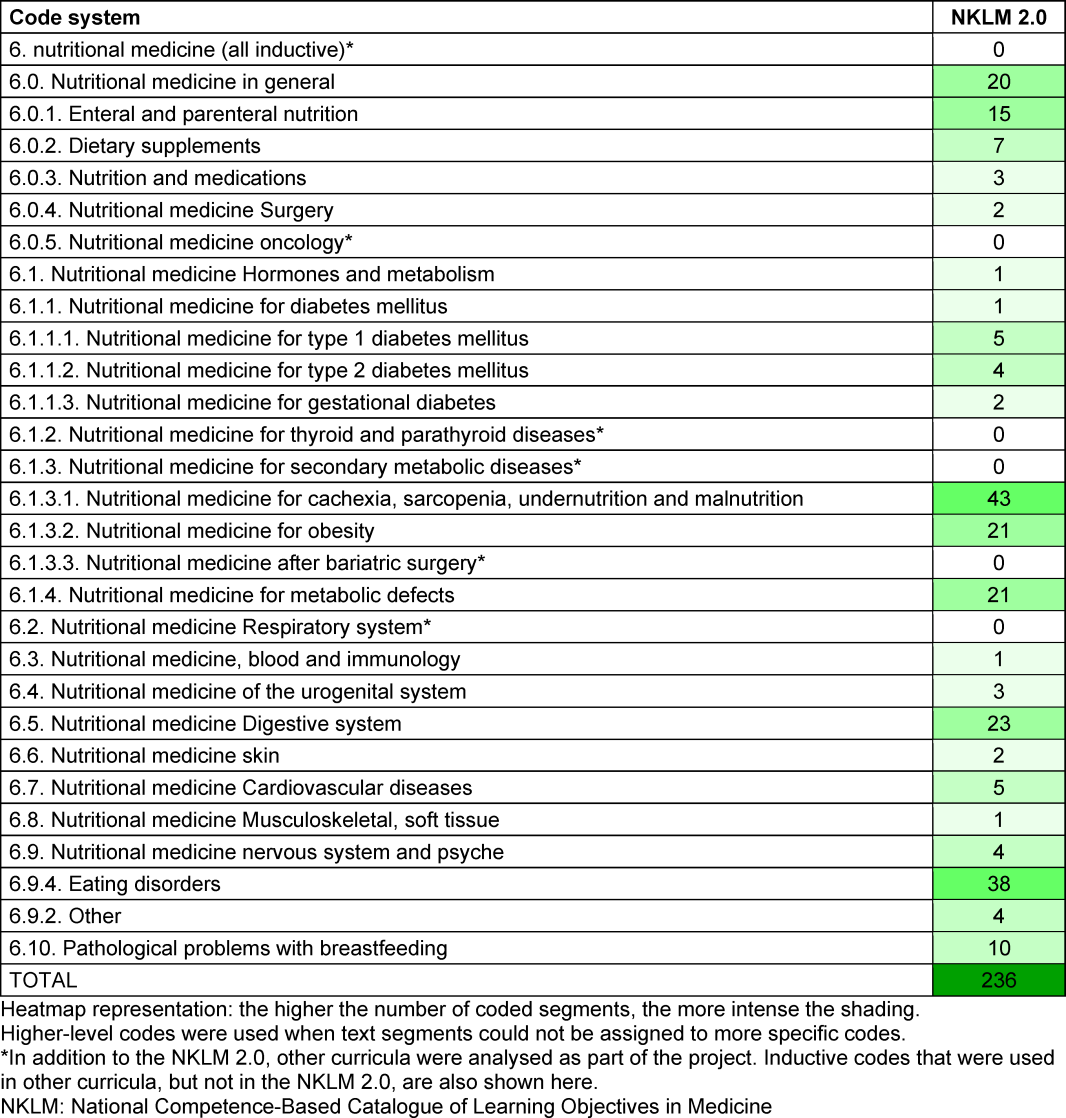
Number of segments coded per code in the NKLM 2.0 in the code category “6. Nutritional medicine”
