# Supplementary material for: Competencies for medical nutritional counselling of children and adolescents: Analysis of NKLM 2.0 based on an evidence-based catalogue of criteria
Source: GMS J Med Educ. 2026 Jun 15;43(5):Doc60. doi: 10.3205/zma001854 (PMC13316368; doi:10.3205/zma001854)
Supplement: Literature search strategy [file JME-43-60-s-001.pdf]

Attachment 1: Literature search strategy

Categories of Criteria Catalogue	Sources	Search Terms	Search Period
**Preventive nutritional knowledge**	Official recommendations, including existing guidelines, consensus papers, positions and statements from recognised national and international professional societies such as the German Nutrition Society (Deutsche Gesellschaft für Ernährung e.V.), the Analytical Nutrient Database, the World Health Organization, the German Society for Paediatrics and Adolescent Medicine (Deutsche Gesellschaft für Kinder- und Jugendmedizin e.V.), and the Federal Centre for Nutrition (Bundeszentrum für Ernährung)<br><br>PubMed, Scopus	nutrition*, eat*, intake*, doctor*, physician*, pediatric*, fat*, protein*, vegetables*	2010-2023
**Communication competencies**	PubMed, Scopus	skill*, communicat*, doctor*, physician*, pediatric*	2013-2023
**Nutrition counselling competencies**	PubMed, Scopus	Effective counsel* method, Effective counsel* pediatric, Effective counsel* diet, Effective counsel* lifestyle, Successful* lifestyle change, Lifestyle change pediatric*, Prevent* obes* pediatric*	2003-2023
**Contextual factors**	PubMed	barrier* diet* counsel*, barrier* diet* counsel* pediatric*	2003-2023

PubMed: database of the National Institutes of Health (NIH)

Attachment 1 to Rudolf SL, Bunzel C, Dietz LM, Kröller K, Markert J, Schörghofer HC, Meixner M, Von Iven L, Lux A, Rissmann A. *Competencies for medical nutritional counselling of children and adolescents: Analysis of NKLM 2.0 based on an evidence-based catalogue of criteria.* GMS J Med Educ. 2026;43(5):Doc60. DOI: 10.3205/zma001854
