# Supplementary material for: Competencies for medical nutritional counselling of children and adolescents: Analysis of NKLM 2.0 based on an evidence-based catalogue of criteria
Source: GMS J Med Educ. 2026 Jun 15;43(5):Doc60. doi: 10.3205/zma001854 (PMC13316368; doi:10.3205/zma001854)
Supplement: Theoretical catalogue of criteria with assigned codes and code memos [file JME-43-60-s-002.pdf]

Attachment 2: Theoretical catalogue of criteria with assigned codes and code memos

Structure point	Overarching learning objective	Learning objective	References	Code system	Code memo
**1.**	**Preventive nutritional knowledge**			1. preventive nutritional knowledge	Use code if no subcode applies and “nutrition” is mentioned only in general terms in the curriculum.
**1.1.**	**The basics of a healthy diet**			1. preventive nutritional knowledge > 1.1. basics of healthy diet	Use code if no subcode is suitable.
1.1.1.	*Ability to assess the benefits and limitations of nutrition for a healthy body in order to support adequate physical and mental development.*			1. preventive nutritional knowledge > 1.1. basics of healthy diet > 1.1.1. nutrient importance, digestion and absorption in general	Benefits/limitations of nutrition for a healthy body and supporting adequate physical/mental development. Importance of nutrition for primary prevention. Anatomy and physiology of digestive tract, microbiome, digestion/ absorption/metabolism of nutrients in general
1.1.1.1.		**Understand**: Basic anatomy and physiology of the digestive system (including microbiome) of children and adolescents	[1-4]		
1.1.1.2.		**Understand**: Basic biochemistry, especially digestion, absorption and metabolism of nutrients in children and adolescents	[1,5]		
1.1.1.3.		**Understand**: Importance of a balanced diet for the primary prevention of various nutrition-associated diseases	[6-15]		
1.1.1.5.		**Understand**: Effects of nutrition on age-appropriate cognitive development	[16-26]		
1.1.1.6.		**Understand**: Effects of nutrition on age-appropriate psychological development	[20-23,27-31]		
1.1.1.7.		**Understand**: Effects of nutrition on age-appropriate physical development	[32,33]		
1.1.1.8.		**Apply:** Recognise and implement individual limits of general nutritional recommendations	[34-42]		

Attachment 2 to Rudolf SL, Bunzel C, Dietz LM, Kröller K, Markert J, Schörghofer HC, Meixner M, Von Iven L, Lux A, Rissmann A. *Competencies for medical nutritional counselling of children and adolescents: Analysis of NKLM 2.0 based on an evidence-based catalogue of criteria.* GMS J Med Educ. 2026;43(5):Doc60. DOI: 10.3205/zma001854

Structure point	Overarching learning objective	Learning objective	References	Code system	Code memo
1.1.2.	*Ability to differentiate and evaluate the characteristics of different types of diets.*			1. preventive nutritional knowledge > 1.1. basics of healthy diet > 1.1.2. different types of diets	Use code when no subcode fits and the curriculum addresses different dietary patterns, types, or forms in general, including their specific characteristics and, where applicable, their advantages and disadvantages. Relevant terms may include: fad diets, special diets, vegetarianism, veganism, cultural dietary practices, religious dietary rules, dietary trends, or dieting
1.1.2.1.		**Understand**: Differences and characteristics of vegetarianism, veganism and subtypes	[43-47]; [https://www.dge.de/wissenschaft/fachinformationen/flexitarier-die-flexiblen-vegetarier/, last reviewed on 23/01/2025]		
1.1.2.2.		**Understand**: Cultural differences in food choices and the impact on the family diet	[48-50]		
1.1.2.3.		**Understand**: Dietary trends/diets among children and adolescents and the influence of (digital) media	[49,51-58]		
1.1.2.4.		**Understand**: Differentiation between medical necessity and the voluntary adoption of a specific/ restrictive diet	[59-64]		
1.1.3.	*Ability to assess nutritional status and prescribe general management and dietary recommendations in accordance with relevant guidelines.*			1. preventive nutritional knowledge > 1.1. basics of healthy diet > 1.1.3. nutritional assessment	Nutritional assessment, dietary history, assessment of dietary behaviour, eating habits, assessment of nutritional status, body mass index, body shape, body height, waist-to-hip ratio, waist circumference, abdominal circumference, calculation of energy requirements, estimation of portion sizes
1.1.3.1.		**Apply:** Routinely carry out and assess a simple nutritional assessment to identify possible risk factors in the nutritional behaviour of children and adolescents	[33,63,65-69]		
1.1.3.2.		**Apply:** Recognise and classify deviations in appearance (body shape and size) from the norm	[66,70-72]		
1.1.3.3.		**Apply:** Calculate and estimate energy requirements according to the paediatric stage of life	[73,74]		

Structure point	Overarching learning objective	Learning objective	References	Code system	Code memo
1.1.3.4.		**Apply:** Assess and evaluate the daily portion sizes consumed in different stages of life in relation to the overall nutritional status	[75-79]		
1.1.3.5.		**Apply:** Access and use guidelines and official nutritional recommendations	[36,80-88]		
1.1.4.	*Ability to recognise the need for interdisciplinary care and to promote collaboration.*			1. preventive nutritional knowledge > 1.1. basics of healthy diet > 1.1.4. interdisciplinary care	This code addresses interdisciplinary care, for example by sensitising physicians, medical assistants and other healthcare professionals to the importance of interdisciplinary collaboration. It includes awareness of the various professional groups involved (e.g. dietitians/nutritionists, physiotherapists, psychotherapists), enabling healthcare professionals to take active steps, assessing when it is appropriate to involve a specialised professional, initiating interdisciplinary care, and motivating patients to make use of these services
1.1.4.1.		**Apply:** Interdisciplinary care leads to greater counselling success (e.g. behavioural change or improved laboratory parameters). Be familiar with the possibilities of interdisciplinary care and promote its expansion and utilisation, e.g. involving nutrition professionals, psychological professionals, social workers and physiotherapists.	[89-93]		
1.1.4.2.		**Apply:** Identify and involve an appropriate specialised professional once recommendations extend beyond general primary prevention.	[85,89,94-102]		
**1.2.**	**Food science**			1. preventive nutritional knowledge > 1.2. food science	
1.2.1.	*Ability to assess and recommend food groups in terms of their nutritional and physiological importance.*			1. preventive nutritional knowledge > 1.2. food science > 1.2.1. foods, food groups, degree of processing	This concerns food groups and foods, as well as the importance of unprocessed and processed foods in the context of primary prevention.
1.2.1.1.		**Apply:** Name and categorise the different food groups consumed in daily diets	[75,79,103,104]		
1.2.1.2.		**Understand**: Holistic understanding of the constituents of foods	[10,75,79,105-108]		

Structure point	Overarching learning objective	Learning objective	References	Code system	Code memo
1.2.1.3.		**Apply:** Know and recommend (nutritionally) equivalent alternatives to foods that are avoided in the family diet	[103,109]		
1.2.1.4.		**Understand**: Importance of unprocessed and processed foods for primary preventive health in childhood and adolescence	[108,110]		
1.2.2.	*Ability to assess the use of supplements and prescribe them individually.*			1. preventive nutritional knowledge > 1.2. food science > 1.2.2. supplements	Use code when the topic concerns supplements or dietary supplements and their use in a primary preventive context (e.g. vitamin B12 intake in a vegan diet). Dietary supplements in nutritional medicine are assigned a separate code.
1.2.2.1.		**Apply:** Recommend nutritional supplements according to life stage (e.g. increased requirement) and current intake (e.g. a special diet)	[111-117]		
1.2.2.2.		**Apply:** Know and assess food supplements according to type (chemical compound) and dosage form (multivitamin preparations, single-dose supplements, tablet, oil, powder)	[117-119]		
1.2.2.3.		**Apply:** Make individualised recommendations regarding the dosage and use of nutritional supplements	[115,116,120]		
1.2.3.	*Ability to provide guidance on food hygiene and toxicology.*			1. preventive nutritional knowledge > 1.2. food science > 1.2.3. food hygiene and toxicology	Hygienic handling of foods, including washing, storage and preparation (e.g. of infant formula), also with regard to the prevention of food-borne infections and intoxications.
1.2.3.1.		**Understand**: Guidance on hygienic practices in the preparation of infant formula and foods	[121-125]; [https://https://www.bfr.bund.de/en/publications/opinions-and-communications/, last reviewed on 30/01/2025]		
1.2.3.2.		**Apply:** Provide guidance on the prevention of food-borne infections and intoxications in specific stages of life	[126-130]		
1.2.3.3.		**Understand**: The impact of inadequate food hygiene on short- and long-term gut health	[2-4,121,122, 127,128, 131]		

Structure point	Overarching learning objective	Learning objective	References	Code system	Code memo
1.2.4.	*Ability to provide guidance on gentle cooking and kitchen techniques.*				
1.2.4.1.		**Apply:** Know and apply gentle cooking methods (to preserve nutrients) and techniques that enhance nutrient availability in order to ensure adequate nutrient intake across different stages of life	[89,109,132-135]		
1.2.4.2.		**Apply:** Communicate the importance of cooking and kitchen techniques, as well as shared meal preparation, for promoting a more balanced food selection among children and adolescents.	[136,137]	1. preventive nutritional knowledge > 1.2. food science > 1.2.4. cooking and kitchen techniques	Cooking and kitchen techniques, preparation of foods and meals, nutrient-preserving preparation methods, shared cooking, etc.
1.2.4.3.		**Apply:** Advise on alternative cooking and kitchen techniques depending on specific life circumstances (e.g. saving time, reducing costs, or accommodating storage possibilities)	[106,137,138]		
**1.3.**	**Nutritional science**			1. preventive nutritional knowledge > 1.3. nutritional science	This section deals with nutrients, their functions, importance, sources, etc. Code 1.3 may be used if none of the more specific codes listed below apply.
1.3.1.	*Ability to assess the macronutrient **protein** in the context of a diet that meets nutritional requirements.*				
1.3.1.1.		**Understand**: The function of proteins and their importance in the body.		1. preventive nutritional knowledge > 1.3. nutritional science > 1.3.1. dietary proteins	Use code when the topic concerns proteins in human nutrition, including sources, requirements, intake recommendations, and metabolism.
1.3.1.2.		**Understand**: Sources of protein, including in meat-free diets (including basic knowledge of biological value)			
1.3.1.3.		**Understand**: Protein requirements across different stages of life	[139-153]		
1.3.1.4.		**Apply:** Recognise signs and symptoms of deficiency or excess of protein or essential amino acids			

Attachment 2 to Rudolf SL, Bunzel C, Dietz LM, Kröller K, Markert J, Schörghofer HC, Meixner M, Von Iven L, Lux A, Rissmann A. *Competencies for medical nutritional counselling of children and adolescents: Analysis of NKLM 2.0 based on an evidence-based catalogue of criteria.* GMS J Med Educ. 2026;43(5):Doc60. DOI: 10.3205/zma001854

Struc-ture point	Overarching learning objective	Learning objective	References	Code system	Code memo
1.3.1.5.		**Apply:** Assess an unfavourable food selection or eating habits that deviate from the norm in the context of protein intake			
1.3.2.	*Ability to assess the macronutrient **fat** in the context of a diet that meets nutritional requirements.*			1. preventive nutritional knowledge > 1.3. nutritional science > 1.3.2. dietary fats	Use code when the topic concerns fats/lipids in human nutrition, including sources, requirements, intake recommendations, and metabolism.
1.3.2.1.		**Understand**: The function of fats and their importance in the body	[26,139,152,154-169]; [https://www.dge.de/wissenschaft/fachinformationen/trans-fettsaeuren-und-die-gesundheit/, last reviewed on 30/01/2025]		
1.3.2.2.		**Understand**: Sources of fat, particularly omega-3 fatty acids			
1.3.2.3.		**Understand**: Requirements for fat, particularly omega-3 fatty acids, across different stages of life			
1.3.2.4.		**Understand**: The importance of omega-3 fatty acids for development			
1.3.2.5.		**Apply:** Recognise signs and symptoms of deficiency or excess of fat, particularly omega-3 fatty acids			
1.3.2.6.		**Apply:** Assess an unfavourable food selection or eating habits that deviate from the norm in the context of fat intake (quantity) and the choice of fat sources (fatty acid profile)			
1.3.3.	*Ability to assess the macronutrient **carbohydrate** in the context of a diet that meets nutritional requirements.*			1. preventive nutritional knowledge > 1.3. nutritional science > 1.3.3. carbohydrates	Use code when the topic concerns carbohydrates, sugars, or dietary fibre in human nutrition, including sources, requirements, intake recommendations, and metabolism.
1.3.3.1.		**Understand**: The function of carbohydrates and their importance in the body	[139,152,167,169-177]; [https://www.dge.de/wissenschaft/fachinformationen/pseudo-getreide-in-der-saeuglings-und-kleinkindernaehrung/, last reviewed on 31/01/2025]		
1.3.3.2.		**Understand**: Sources of carbohydrates, particularly sources of dietary fibre			
1.3.3.3.		**Understand**: Requirements for carbohydrates and dietary fibre across different stages of life			

Structure point	Overarching learning objective	Learning objective	References	Code system	Code memo
1.3.3.4.		**Apply:** Recognise signs and symptoms of deficiency or excess of carbohydrates (particularly dietary fibre and sugar)			
1.3.3.5.		**Apply:** Assess an unfavourable food selection or eating habits that deviate from the norm in the context of carbohydrate intake (particularly dietary fibre and sugar)			
1.3.4.	*Ability to assign potentially critical **micronutrients** to different stages of life and assess them in the context of a diet that meets nutritional requirements.*			1. preventive nutritional knowledge > 1.3. nutritional science > 1.3.4. micronutrients	Use code when the topic concerns micronutrients in human nutrition (vitamins, minerals, macro- and trace elements), including sources, requirements, intake recommendations, and metabolism.
1.3.4.1.		**Understand**: Identify potentially critical micronutrients across different stages of life.	[107,178-182]		
1.3.4.2.		**Understand**: The function and importance of potentially critical micronutrients for health	[107,137,139,152,166, 174,178-187]; [https://www.dge.de/wissenschaft/fachinformationen/sekundaere-pflanzenstoffe-und-die-gesundheit/, last reviewed on 31/02/2025]		
1.3.4.3.		**Understand**: Sources of potentially critical micronutrients			
1.3.4.4.		**Understand**: Micronutrient requirements across different stages of life			
1.3.4.5.		**Apply**: Recognise signs and symptoms of deficiency/oversupply of potentially critical micronutrients			
1.3.4.6.		**Apply:** Assess unfavourable food selection or eating habits that deviate from the norm in the context of appropriate micronutrient sources			
1.3.5.	*Ability to assign **fluid requirements** to life stages and recommend adequate intake.*			1. preventive nutritional knowledge > 1.3. nutritional science > 1.3.5. fluid	Use code when the topic concerns fluid intake in human nutrition, including sources, requirements, and intake recommendations.

Structure point	Overarching learning objective	Learning objective	References	Code system	Code memo
1.3.5.1.		**Understand**: Knowledge of the function and importance of adequate fluid intake for health	[139,152,172,177, 188-198]		
1.3.5.2.		**Understand**: Sources/selection of appropriate beverages to meet fluid requirements			
1.3.5.3.		**Understand**: Knowledge of fluid requirements across different stages of life			
1.3.5.4.		**Apply**: Recognise signs and symptoms of a deficiency/excess related to (unfavourable) drinks			
1.3.5.5.		**Apply**: Assess an unfavourable beverage selection or drinking habits that deviate from the norm			
1.3.6.	*Ability to provide current and individual recommendations on exclusive breastfeeding, complementary feeding, and the transition to the family diet.*				
1.3.6.1.		**Understand:** The time window of the first 1,000 days as a sensitive period of life (specifically in the context of nutrition and the health of mother and child)	[121,199-203]	1. preventive nutritional knowledge > 1.3. nutritional science > 1.3.7. breastfeeding	Use code when the topic concerns breastfeeding in general, for example when the term breastfeeding is mentioned alone, as well as when referring to the manner of breastfeeding, such as frequency, duration, or latching technique.
				1. preventive nutritional knowledge > 1.3. nutritional science > 1.3.7. breastfeeding > 1.3.7.1. infant nutrition	Use code when the topic concerns the infant's supply of nutrients, immunoglobulin A, etc., via breast milk and/or the use of infant formula.
				1. preventive nutritional knowledge > 1.3. nutritional science > 1.3.8. complementary feeding	Use code when the topic concerns complementary feeding, e.g. the introduction of complementary foods, their composition, preparation, etc. No more detailed codes will be provided.

Struc-ture point	Overarching learning objective	Learning objective	References	Code system	Code memo
				1. preventive nutritional knowledge > 1.3. nutritional science > 1.3.10. toddler nutrition	Use code when the topic concerns the nutrition of toddlers.
1.3.6.2.		**Understand**: The importance of exclusive and predominant breastfeeding during the first six months of an infant's life		1. preventive nutritional knowledge > 1.3. nutritional science > 1.3.7. breastfeeding	Use code when the topic concerns breastfeeding in general, for example when the term breastfeeding is mentioned alone, as well as when referring to the manner of breastfeeding, such as frequency, duration, or latching technique.
1.3.6.3.		**Understand**: The influence of the breastfeeding person's nutrient intake on milk production and milk quality	[201,204-209]	1. preventive nutritional knowledge > 1.3. nutritional science > 1.3.7. breastfeeding > 1.3.7.2. nutrition of breastfeeding mothers	Use code when the topic concerns the mother's diet during the breastfeeding period.
1.3.6.4.		**Apply**: Advise caregivers on the conditions of breastfeeding recommendations, such as duration of breastfeeding, feeding rhythm, and latching technique	[200-202,209-211]	1. preventive nutritional knowledge > 1.3. nutritional science > 1.3.7. breastfeeding	Use code when the topic concerns breastfeeding in general, for example when the term breastfeeding is mentioned alone, as well as when referring to the manner of breastfeeding, such as frequency, duration, or latching technique.
1.3.6.5.		**Apply:** Identify the necessity and timing of introducing infant formula as a supplement (partial breastfeeding) or as the sole feeding method (e.g. in cases of infant weight loss)	[200,201,210]	1. preventive nutritional knowledge > 1.3. nutritional science > 1.3.7. breastfeeding > 1.3.7.1. infant nutrition	Use code when the topic concerns the infant's supply of nutrients, immunoglobulin A, etc., via breast milk and/or the use of infant formula.
1.3.6.6.		**Understand**: Individualised dietary recommendations during the breastfeeding period (in cases of persistent symptoms in the breastfed infant)	[200,210-212]	1. preventive nutritional knowledge > 1.3. nutritional science > 1.3.7. breastfeeding > 1.3.7.3. challenges	Use code when the topic concerns challenges related to breastfeeding that are not of a pathological nature.
1.3.6.7.		**Apply**: Advise caregivers on individualised recommendations for the adapted introduction of complementary feeding (transition from exclusive to partial breastfeeding)	[121,201,213,214]	1. preventive nutritional knowledge > 1.3. nutritional science > 1.3.8. complementary feeding	Use code when the topic concerns complementary feeding, e.g. the introduction of complementary foods, their composition, preparation, etc. No more detailed codes will be provided.
1.3.6.8.		**Understand**: The importance of primary preventive education for caregivers regarding a balanced family diet from the first year of life onward	[120,201,215]	1. preventive nutritional knowledge > 1.3. nutritional science > 1.3.10. toddler nutrition	Use code when the topic concerns the nutrition of toddlers.

Structure point	Overarching learning objective	Learning objective	References	Code system	Code memo
1.3.6.9.		**Apply**: Assess an unfavourable food selection or eating habits that deviate from the norm in the context of exclusive breastfeeding, partial breastfeeding, and the family diet phase	[187,216-221]	1. preventive nutritional knowledge > 1.3. nutritional science > 1.3.8. complementary feeding	Use code when the topic concerns complementary feeding, e.g. the introduction of complementary foods, their composition, preparation, etc. No more detailed codes will be provided.
				1. preventive nutritional knowledge > 1.3. nutritional science > 1.3.10. toddler nutrition	Use code when the topic concerns the nutrition of toddlers.
				1. preventive nutritional knowledge > 1.3. nutritional science > 1.3.9. maternal nutrition during pregnancy (inductive)	Use code when the topic concerns nutrition during pregnancy. All related content should be grouped under this code; no more detailed codes will be created.
**1.4.**	**Sociology and psychology of nutrition**			1. preventive nutritional knowledge > 1.4 Sociology and psychology of nutrition	Use code when none of the subcodes apply, but the topic concerns the sociology and psychology of nutrition, e.g. eating behaviour, prevention of eating disorders, or the influence of sociocultural factors on eating and dietary behaviour.
1.4.1.	*Ability to contextualise eating behaviour and assess it appropriately.*			1. preventive nutritional knowledge > 1.4. sociology and psychology of nutrition > 1.4.1. eating behaviour	Use code when the topic concerns eating behaviour in general. Apply it when eating behaviour is mentioned as a keyword without further details that would correspond to the more specific codes.
1.4.1.1.		**Understand**: Knowledge of the development of eating behaviour and influencing factors in the context of the family diet	[222-226]		
1.4.1.2.		**Apply**: Know and assess contextual factors (e.g. socioeconomic status) that may influence dietary behaviour across different stages of life.	[89,227-231]		
1.4.1.3.		**Apply**: Know and assess contextual factors (e.g. stress, family, peer group) that may influence dietary behaviour across different stages of life.	[89,222,225,227, 232-236]		

Structure point	Overarching learning objective	Learning objective	References	Code system	Code memo
1.4.1.4.		**Apply**: Recognise and assess problematic eating behaviour across different stages of life	[237-240]	1. preventive nutritional knowledge > 1.4. sociology and psychology of nutrition > 1.4.1. eating behaviour > 1.4.1.4. problematic eating behaviour	Use code when dealing with problematic eating behaviour that is still considered subclinical (not symptoms of eating disorders).
1.4.1.5.		**Apply**: Recognise and assess the various symptoms of eating disorders and the associated risks	[241-243]	1. preventive nutritional knowledge > 1.4. sociology and psychology of nutrition > 1.4.1. eating behaviour > 1.4.1.5. symptoms of eating disorders	Use code when the topic concerns symptoms of eating disorders and the associated risks. This may also be understood in terms of early detection (strictly speaking secondary prevention). All other aspects of eating disorders, particularly therapeutic aspects, can be found in the nutrition medicine section.
				1. preventive nutritional knowledge > 1.5. nutritional knowledge for the primary prevention of specific diseases (inductive)	Use code when the topic concerns specific nutritional knowledge related to particular diseases. The prevention of food-borne diseases (infection, intoxication) is coded under 1.2.3.
				1. preventive nutritional knowledge > 1.6. lifestyle without specific nutritional reference (inductive)	Use code when the curriculum refers to lifestyle or way of life without explicitly mentioning nutrition.
**2.**	**Communication**			**2. communication competencies**	Use code if only general communication is mentioned, but no further details are provided.
2.1.	*Ability to ensure patient- and family-centred communication and care.*			2. communication competencies > 2.1 Patient- and family-centred care	Use code if patient- and/or family-centred care is explicitly mentioned.
2.1.1.		**Understand**: Basic attitude: advantages and aspects of patient- and family-centred communication and care (in contrast to provider-centred communication).	[89,244-261]; Specific to empathy: [256,262-264]		

Struc-ture point	Overarching learning ob-jective	Learning objective	References	Code system	Code memo
2.1.2.		**Apply**: Patient- and family-centred care:<br>- Empathy<br>- Active listening<br>- Validation<br>- Comprehensible explanations<br>- Shared decision-making<br>- Respecting the patient's autonomy<br>- Building a relationship and trust<br>- Activating patients to actively partici-pate in and shape their treatment			
2.1.4.		**Apply**: Coping with difficult situations (e.g. conveying "bad news", conflict, but also risk communication in medi-cal consultation), requires e.g. skills for emotion regulation, de-escalation, empathy, change of perspective	[259,265-270]	2. communication compe-tencies > 2.1 Patient- and family-centred care > 2.1.4 Dealing with difficult situa-tions	Dealing with difficult situations (e.g. convey-ing "bad news", conflict, but also risk com-munication medical consultation), requires e.g. skills for emotion regulation, de-escala-tion, empathy, change of perspective
2.1.5.		**Apply**: Intercultural competence, e.g. knowledge regarding understanding of roles, eating habits, influence of reli-gion, professional handling of lan-guage barriers	[259,271-277]	2. communication compe-tencies > 2.1 Patient- and family-centred care > 2.1.5 Intercultural competencies	Intercultural competence, e.g. knowledge re-garding understanding of roles, eating hab-its, influence of religion, professional handling of language barriers
2.2.	*Ability to understand and apply special features of communication in paediat-rics.*			2. communication compe-tencies > 2.2 Communica-tion in paediatrics	Use code when communication in paediat-rics is specifically addressed. This can mean adapting communication to age and stage of development, addressing children and par-ents, building relationships, encouraging family meals, activating fathers
2.2.1.		**Apply**: Ability to adapt one's commu-nication to age and stage of develop-ment.	[244,278-280]		
2.2.2.		**Apply**: Ability to address and involve both children and their parents, build relationships	[244,278,279, 281-292]		
2.2.3.		**Apply**: Ability to assess the family sit-uation, promote family meals, activate fathers/partners, especially with re-gard to breastfeeding	[226,293-301]		
				2. communication compe-tencies > 2.3. communica-tion: other (inductive)	Section 2.3. provides space for inductively created codes regarding communication.

Struc-ture point	Overarching learning objective	Learning objective	References	Code system	Code memo
				2. communication competencies > 2.3. communication: other (inductive) > 2.3.1. transmitter-receiver model (inductive)	Transmitter-receiver model
				2. communication competencies > 2.3. communication: other (inductive) > 2.3.2. four-ears model (inductive)	Four-ears model, four-ears-four-beaks, four sides of a message, Schulz von Thun
				2. communication competencies > 2.3. communication: other (inductive) > 2.3.3. non-violent communication (inductive)	Non-violent communication, Marshall Rosenberg,
**3.**	**Nutrition counselling competencies**			3. nutrition counselling competencies	
3.1.	*Ability to apply counselling psychology methods.*			3. nutrition counselling competencies > 3.1 Counselling psychological methods	
3.1.1.		**Understand**: Transtheoretical model (of behavioural change, according to Prochaska and DiClemente)	[302-304]	3. nutrition counselling competencies > 3.1 Counselling psychological methods > 3.1.1 Transtheoretical model of behaviour change	Transtheoretical model (of behavioural change, according to Prochaska and DiClemente)
3.1.2.		**Apply**: Knowledge of and ability to apply Motivational Interviewing, in consultations with parents, children as well as adolescents	[89,261,287-289, 305-315]	3. nutrition counselling competencies > 3.1 Psychological counselling methods > 3.1.2 Motivational interviewing	Knowledge and ability to apply Motivational Interviewing, with parents, children, and adolescents. The term must be explicitly identified: Motivational Interviewing.

Structure point	Overarching learning objective	Learning objective	References	Code system	Code memo
3.1.3.		**Apply**: Knowledge and ability to apply counselling psychology methods to promote behavioural change:<br><br>- Agree on realistic goals with patients<br>- Guide self-monitoring<br>- Positive reinforcement, e.g. by providing feedback<br>- Plan longer-term collaboration and progress monitoring (e.g. using self-monitoring)	[89,261,308, 315-323]	3. nutrition counselling competencies > 3.1 Counselling psychological methods > 3.1.3 Goal-setting and self-monitoring	Knowledge and ability to apply psychological counselling methods to promote behavioural change:<br>- Agree on realistic goals with patients<br>- Guide patients to self-monitoring<br>- Positive reinforcement, e.g. by providing feedback<br>- Plan longer-term collaboration and progress monitoring (e.g. using self-monitoring). Use code if at least goal setting and/or self-monitoring are mentioned (in the sense of "tracking").
3.1.4.		**Apply:** Resource- and solution-oriented counselling, especially for action and coping planning (relapse prevention)	[308,316,324-327]	3. nutrition counselling competencies > 3.1. psychological counselling methods > 3.1.4. resource- and solution-oriented communication	Resource- and solution-oriented communication, especially for action and coping planning (relapse prevention). Use code if at least one of the terms is mentioned: Resource-oriented or resource-activating communication and/or solution-oriented or solution-focused counselling.
3.1.5.		**Apply:** Know the benefits and limitations of nudging. Advise on the use of nudging within the family environment.	[89,328,329]	3. nutrition counselling competencies > 3.1. psychological counselling methods > 3.1.5. nudging	Know the advantages and limits of nudging. Advise on the use of nudging within the family environment.
3.1.6.		**Understand:** Increased eating disorder risk due to dieting and obesity treatment, particularly for a vulnerable subgroup of people.	[330-338]	3. nutrition counselling competencies > 3.1 Psychological counselling methods > 3.1.6 Diets and the risk of eating disorders	Increased risk of developing eating disorders due to dieting and obesity treatment, particularly for a vulnerable subgroup of people. Use code when, at least theoretically, the connection between dieting and the risk of eating disorders is discussed. This fact should be taken into account in counselling on diet and exercise. Nutritional counselling should always include the promotion of mental health and a healthy body image.
3.1.7.		**Apply:** Individual and sensitive approach in counselling regarding body weight to promote mental health and a healthy body image.		3. nutrition counselling competencies > 3.1 Psychological counselling methods > 3.1.7 sensitive communication about body weight	Individual and sensitive approach in body weight counselling to promote mental health and a healthy body image. Use the code when practising sensitive handling is explicitly indicated.

Structure point	Overarching learning objective	Learning objective	References	Code system	Code memo
3.2.	*Ability to use appropriate materials in counselling.*			3. nutrition counselling competencies > 3.2 Use of counselling materials	Ability to utilize suitable counselling materials, such as information material or graphic illustrations, to supplement counselling. This includes material such as a plate model or food pyramid, as well as digital applications.
3.2.1.		**Apply:** Ability to utilize suitable counselling materials, such as information material or graphic illustrations, to supplement counselling	[84,279,339,340]		
3.2.2.		**Understand**: Know the possibilities, limits and risks of using digital applications and forms of communication with regard to changing nutritional and eating behaviour.	[89,315,341-347]		
3.3.	*Ability to recognise the importance of self-care and self-efficacy of the healthcare professional.*			3. nutrition counselling competencies > 3.3 Self-care, self-efficacy of the professional	Use code when it is generally addressed that the health professional's self-efficacy expectation is important for opening a conversation about nutrition in the first place. Background: This increases the likelihood that diet/lifestyle factors will be addressed by physicians at all. It also acts as a mediating factor for successful counselling, e.g. by promoting participatory counselling and leading to greater patient satisfaction. The role model function of doctors is an important factor in relation to nutrition
3.3.1.		**Apply:** Adequate self-efficacy expectation of the counselling health professional.	[84,132,133, 348-355]		
3.3.2.		**Understand:** Healthcare professional has an influential role model function with regard to lifestyle factors such as diet and exercise.	[89,132,133]		
				3. nutrition counselling competencies > 3.4. counselling: other (inductive)	Section 3.4. provides space for inductively created codes for counselling.
				3. nutrition counselling competencies > 3.4. counselling: other (inductive) > 3.4.1. systemic counselling (inductive)	Relevant terms: systemic counselling, systemic counselling, systemic therapy, systemic attitude, systemics, systemic questioning techniques
				3. nutrition counselling competencies > 3.4. counselling: other (inductive) > 3.4.2. biopsychosocial model (inductive)	Biopsychosocial model

Structure point	Overarching learning objective	Learning objective	References	Code system	Code memo
				3. nutrition counselling competencies > 3.4. counselling: other (inductive) > 3.4.3. other models of health behaviour (inductive)	Health psychology models that aim to represent health behaviour or (lack of) behaviour change, such as the Health Action Process Approach, Rubicon Model, Health Belief Model, Protection Motivation Theory, Theory of Planned Behaviour, Social Cognitive Theory
**4.**	**Nutrition communication through the use of written materials (such as information leaflets)**				
**5.**	**Contextual factors**				
5.1.		Sufficient time	[84,85,100,352,356]		
5.2.		Sufficient remuneration	[84,85,352,357]		
5.3.		Cooperation with/ access to/ networking with nutritionists	[84,85,89,100,101]		
5.4.		Appropriate documentation software	[84,315,357]		
5.5.		Possibility of regular and long-term support	[279,308,316-320]		
5.6.		Suitable training and further education	[355,358-362]		
				6. nutritional medicine (all inductive)	These are codes added inductively from curricula that do not have a direct primary preventive relation to nutrition.
				6. nutritional medicine > 6.0. nutritional medicine in general	Use code when none of the more specific subcodes listed below apply. The learning objective to be coded in the curriculum may refer to children or adults.
				6. nutritional medicine > 6.0. nutritional medicine in general > 6.0.1. enteral and parenteral nutrition	Use code when none of the more specific subcodes listed below apply, for example because no specific disease is mentioned for which (par)enteral nutrition should be used. The learning objective to be coded in the curriculum may refer to children or adults.
				6. nutritional medicine > 6.0. nutritional medicine in general > 6.0.2. dietary supplements	Dietary supplements in nutritional medicine, e.g. the use of nutrient preparations to compensate for nutrient deficiencies caused by a disease.

Struc-ture point	Overarching learning objective	Learning objective	References	Code system	Code memo
				6. nutritional medicine > 6.0. nutritional medicine in general > 6.0.3. nutrition and medications	e.g. interactions between foods/nutrients and medications.
				6. nutritional medicine > 6.0. nutritional medicine basics > 6.0.4. nutritional medicine surgery	Nutrition before and after surgery (except bariatric surgery; see: endocrinology). Use code only if none of the more specific subcodes listed below apply, for example if no specific disease is mentioned for which nutritional medicine should be applied. The learning objective to be coded in the curriculum may refer to children or adults.
				6. nutritional medicine > 6.0. nutritional medicine in general > 6.0.5. nutritional medicine oncology	Use code when the topic concerns any form of nutrition therapy in the context of oncological diseases, either during or after treatment. Exception: do not use code for nutrition therapy following surgical procedures due to oncological diseases (e.g. status post gastrectomy).
				6. nutritional medicine > 6.1 Hormones and metabolism	Use code when none of the more specific subcodes listed below apply. The learning objective to be coded in the curriculum may refer to children or adults. A clear relation to nutrition must be present, which may be indicated by terms such as nutrition, carbohydrates, nutrient intake, nutritional counselling, dietetics, etc.
				6. nutritional medicine > 6.1. hormones and metabolism > 6.1.1. nutritional medicine for diabetes mellitus	Use code when none of the more specific subcodes listed below apply. The learning objective to be coded in the curriculum may refer to children or adults. A clear relation to nutrition must be present, which may be indicated by terms such as nutrition, carbohydrates, nutrient intake, nutritional counselling, dietetics, etc.

Struc-ture point	Overarching learning objective	Learning objective	References	Code system	Code memo
				6. nutritional medicine > 6.1. hormones and metabolism > 6.1.1. diabetes mellitus > 6.1.1.1. nutritional medicine for type 1 diabetes mellitus	Use code when the curriculum clearly refers to nutritional aspects of type 1 diabetes mellitus, such as the amount and quality of carbohydrates, adjusting medication to nutrition and vice versa, etc. It may refer to children or adults.
				6. nutritional medicine > 6.1. hormones and metabolism > 6.1.1. diabetes mellitus > 6.1.1.2. nutritional medicine for type 2 diabetes mellitus	Use code when the curriculum clearly refers to nutritional aspects of type 2 diabetes mellitus, such as the amount and quality of carbohydrates, adjusting medication to nutrition and vice versa, etc. It may refer to children or adults.
				6. nutritional medicine > 6.1. hormones and metabolism > 6.1.1. diabetes mellitus > 6.1.1.3. nutritional medicine for gestational diabetes	
				6. nutritional medicine > 6.1. hormones and metabolism > 6.1.2. nutritional medicine for thyroid and parathyroid diseases	Use code when the curriculum clearly refers to nutritional aspects of thyroid or adrenal disorders, such as nutrition in thyroid diseases in general, iodine intake, fluid and electrolyte balance, etc. It may refer to children or adults.
				6. nutritional medicine > 6.1. hormones and metabolism > 6.1.3. nutritional medicine for secondary metabolic diseases	
				6 Nutritional medicine > 6.1 Hormones and metabolism > 6.1.3 Nutritional medicine for secondary metabolic diseases > 6.1.3.1 Nutritional medicine for cachexia, sarcopenia, undernutrition and malnutrition	Nutritional aspects of the development and treatment of conditions such as cachexia in geriatrics, disease-related malnutrition, malignancy-associated cachexia, refeeding syndrome, sarcopenic obesity, undernutrition and malnutrition due to reduced intake of proteins and micronutrients, including pellagra, scurvy, dehydration due to insufficient fluid intake (geriatrics), and funicular myelosis (vitamin B12 deficiency), etc.

Structure point	Overarching learning objective	Learning objective	References	Code system	Code memo
				6. nutritional medicine > 6.1. hormones and metabolism > 6.1.3. nutritional medicine for secondary metabolic diseases > 6.1.3.2. nutritional medicine for obesity	Nutritional aspects of the development, prevention, and treatment of obesity, such as energy intake, quality and degree of processing of foods, beverage selection, vegetable consumption, etc.
				6. nutritional medicine > 6.1. hormones and metabolism > 6.1.3. nutritional medicine for secondary metabolic diseases > 6.1.3.3. nutritional medicine after bariatric surgery	
				6. nutritional medicine > 6.1 Hormones and metabolism > 6.1.4 Nutritional medicine for metabolic defects	Use code for nutritional medicine in conditions such as cystic fibrosis, disorders of amino acid metabolism, disorders of glucose metabolism (e.g. galactosaemia, glycogen storage diseases, hereditary fructose intolerance), hyperuricaemia, gout, alcohol-related diseases, and disorders of trace element metabolism (e.g. haemochromatosis, Wilson's disease).
				6. nutritional medicine > 6.2. nutritional medicine: respiratory system	Use code for nutritional medicine in diseases such as chronic obstructive pulmonary disease.

Structure point	Overarching learning objective	Learning objective	References	Code system	Code memo
				6. nutritional medicine > 6.3. nutritional medicine: blood and immunology	e.g. nutritional medicine in infection with the human immunodeficiency virus, dietary recommendations in acute inflammation of the stomach and intestines, prevention of inflammation of the stomach and intestines through hygiene, prevention of infection-related haemolytic uraemic syndrome (e.g. caused by enterohaemorrhagic Escherichia coli) through food hygiene, severe acute respiratory syndrome coronavirus 2 infection (SARS-CoV-2) and its consequences. Exception: 1.2.3.2 Prevention of food-borne infections and intoxications. Iron deficiency anaemia should be coded as “malnutrition.”
				6. nutritional medicine > 6.4. nutritional medicine: urogenital system	e.g. nutritional medicine in urolithiasis (such as nutrition in or after kidney stones, fluid intake, prevention of kidney stone formation), kidney insufficiency (acute, chronic, haemodialysis), polycystic ovary syndrome, endometriosis.

Struc-ture point	Overarching learning ob-jective	Learning objective	References	Code system	Code memo
				6. nutritional medicine > 6.5. nutritional medicine: di-gestive system	Use code for nutritional medicine in all dis-eases and pathological conditions of the di-gestive system that are not covered by other codes (such as surgery). This includes, for example: nutritional medicine in reflux oe-sophagitis, heartburn, Barrett's oesophagus, gastritis (acute and chronic), gastroduodenal ulcer disease caused by Helicobacter pylori, pyloric stenosis, irritable stomach, functional dyspepsia, food allergies including eosino-philic oesophagitis, food intolerances (such as lactose intolerance, fructose malabsorp-tion, histamine intolerance), irritable bowel syndrome (including intolerance to fermenta-ble oligosaccharides, disaccharides, mono-saccharides and polyols), coeliac disease and its consequences, chronic inflammatory bowel diseases (Crohn's disease, ulcerative colitis), small and large intestinal diverticula, diverticulosis, diverticulitis, constipation, fae-cal incontinence, diseases of the liver, gallbladder, or pancreas, small intestinal bacterial or fungal overgrowth, and chewing and swallowing disorders (in general or with-out specification of the cause).
				6. nutritional medicine > 6.6. nutritional medicine skin	Nutritional medicine in atopic dermatitis, rosacea, psoriasis, etc.

Structure point	Overarching learning objective	Learning objective	References	Code system	Code memo
				6. nutritional medicine > 6.7. nutritional medicine cardiovascular diseases	Nutritional medicine aspects related to the prevention and treatment of: dyslipidaemia, hypercholesterolaemia, hypertriglyceridaemia, disorders of lipid metabolism, arteriosclerosis, atherosclerosis, peripheral arterial occlusive disease, primary or essential hypertension, coronary heart disease (stable or acute), angina pectoris, myocardial infarction, heart attack, status post myocardial infarction, stroke, status post stroke. These conditions have been grouped together because there are major overlaps in aetiology, nutritional medicine, and dietetic management, which cannot be clearly separated.
				6. nutritional medicine > 6.8. nutritional medicine musculoskeletal, soft tissue	Nutritional medicine aspects of inflammatory rheumatic diseases, osteoporosis, osteomalacia, rickets, etc.
				6. nutritional medicine > 6.9. nutritional medicine nervous system and psyche	
				6. nutritional medicine > 6.9. nutritional medicine nervous system and psyche > 6.9.4. eating disorders	Diagnosis and treatment of eating disorders, such as anorexia nervosa, anorexia, bulimia nervosa, bulimia, binge eating, binge eating disorder, avoidant-restrictive food intake disorder, pica, etc. Symptoms and early detection are classified under nutritional psychology in the primary prevention section (1.4.1.5).
				6. nutritional medicine > 6.9. nutritional medicine nervous system and psyche > 6.9.2. other	Nutritional medicine aspects in intoxication and addiction, such as alcohol and drug use, as well as nutritional medicine in somatoform disorders, somatisation disorders, somatoform autonomic dysfunction, epilepsy, epileptic seizures, depression, depressive disorders, and all other disorders of the psyche and/or nervous system that are not attributable to an already listed underlying disease.

Structure point	Overarching learning objective	Learning objective	References	Code system	Code memo
				6. nutritional medicine > 6.10. Pathological problems with breastfeeding	Use code when the topic concerns pathological problems related to breastfeeding, such as breastfeeding refusal. Codes for specific diseases (e.g. pyloric stenosis) already exist under “Nutritional medicine of the digestive system” (6.5).

etc.- et cetera; e.g.- for example
Inductive codes are highlighted in light grey.

References

1. Del Ciampo LA, Del Ciampo IRL. Anatomical and Physiological Characteristics of the Digestive Tract in Childhood. ACRI. 2020:50–56. DOI: 10.9734/acri/2020/v20i330184.
2. Drago L, Panelli S, Bandi C, Zuccotti G, Perini M, D'Auria E. What Pediatricians Should Know Before Studying Gut Microbiota. Journal of clinical medicine. 2019;8(8). DOI: 10.3390/jcm8081206.
3. Goulet O, Hojsak I, Kolacek S, Pop TL, Cokugras FC, Zuccotti G, et al. Paediatricians play a key role in preventing early harmful events that could permanently influence the development of the gut microbiota in childhood. Acta paediatrica (Oslo, Norway : 1992). 2019;108(11):1942–54. DOI: 10.1111/apa.14900.
4. Simon M-C, Sina C, Ferrario PG, Daniel H. Gut Microbiome Analysis for Personalized Nutrition: The State of Science. Molecular nutrition & food research. 2023;67(1):e2200476. DOI: 10.1002/mnfr.202200476.
5. Melse-Boonstra A. Bioavailability of Micronutrients From Nutrient-Dense Whole Foods: Zooming in on Dairy, Vegetables, and Fruits. Frontiers in nutrition. 2020;7:101. DOI: 10.3389/fnut.2020.00101.
6. Verduci E, Bronsky J, Embleton N, Gerasimidis K, Indrio F, Köglmeier J, et al. Role of Dietary Factors, Food Habits, and Lifestyle in Childhood Obesity Development: A Position Paper From the European Society for Paediatric Gastroenterology, Hepatology and Nutrition Committee on Nutrition. Journal of pediatric gastroenterology and nutrition. 2021;72(5):769–83. DOI: 10.1097/MPG.0000000000003075.
7. Daniels SR, Hassink SG. The Role of the Pediatrician in Primary Prevention of Obesity. Pediatrics. 2015;136(1):e275-92. DOI: 10.1542/peds.2015-1558.
8. Mozaffarian D. Dietary and Policy Priorities for Cardiovascular Disease, Diabetes, and Obesity: A Comprehensive Review. Circulation. 2016;133(2):187–225. DOI: 10.1161/CIRCULATIONAHA.115.018585.
9. Lucas A, Sampson HA. Infant nutrition and primary prevention: current and future perspectives. Nestle Nutrition workshop series. Paediatric programme. 2006;57:1–13. DOI: 10.1159/000091023.
10. Koletzko B, Bhatia J, Bhutta ZA, Cooper P, Makrides M, Uauy R, et al. Pediatric Nutrition in Practice, Vol 113: S. Karger AG; 2015.
11. Koletzko B. Ernährung und kindliche Gesundheit. Monatsschr Kinderheilkd. 2022;170(2):114–15. DOI: 10.1007/s00112-021-01415-2.
12. Kersting M, Kalhoff H, Lücke T. Das neue FKE lebt – Kinderernährung und Pädiatrie gehören zusammen. Ernährung & Medizin. 2017;32(01):7–8. DOI: 10.1055/s-0043-103649.
13. Saavedra JM, Dattilo AM. Nutrition in the First 1000 Days of Life: Society's Greatest OpportunityaaThis manuscript serves as the introduction to the book:

Early nutrition and long-term health: Mechanisms, consequences, and opportunities. J. Saavedra & A. Dattilo (Eds.), Elsevier, Oxford. In: Early Nutrition and Long-Term Health: Elsevier; 2017. p. xxxv–xliv.

14. Bhattarai N, Prevost AT, Wright AJ, Charlton J, Rudisill C, Gulliford MC. Effectiveness of interventions to promote healthy diet in primary care: systematic review and meta-analysis of randomised controlled trials. BMC public health. 2013;13:1203. DOI: 10.1186/1471-2458-13-1203.

15. Bührer C, Ensenauer R, Jochum F, Kalhoff H, Körner A, Koletzko B, et al. Standards der ernährungsmedizinischen Versorgung in der ambulanten und stationären Pädiatrie durch spezialisierte Einrichtungen der Kinder- und Jugendmedizin. Monatsschr Kinderheilkd. 2020;168(9):834–41. DOI: 10.1007/s00112-020-00901-3.

16. Nyaradi A, Li J, Hickling S, Foster J, Oddy WH. The role of nutrition in children's neurocognitive development, from pregnancy through childhood. Frontiers in human neuroscience. 2013;7:97. DOI: 10.3389/fnhum.2013.00097.

17. Roberts M, Tolar-Peterson T, Reynolds A, Wall C, Reeder N, Rico Mendez G. The Effects of Nutritional Interventions on the Cognitive Development of Preschool-Age Children: A Systematic Review. Nutrients. 2022;14(3). DOI: 10.3390/nu14030532.

18. Lam LF, Lawlis TR. Feeding the brain - The effects of micronutrient interventions on cognitive performance among school-aged children: A systematic review of randomized controlled trials. Clinical nutrition (Edinburgh, Scotland). 2017;36(4):1007–14. DOI: 10.1016/j.clnu.2016.06.013.

19. Larson L, Yousafzai A. Impact of Nutritional Interventions on Mental Development of Children Under-two in Developing Countries: A Systematic Review of Randomized Controlled Trials. EJNFS. 2015;5(5):549–50. DOI: 10.9734/ejnfs/2015/20958.

20. Rosales FJ, Reznick JS, Zeisel SH. Understanding the role of nutrition in the brain and behavioral development of toddlers and preschool children: identifying and addressing methodological barriers. Nutritional neuroscience. 2009;12(5):190–202. DOI: 10.1179/147683009X423454.

21. Benton D. The influence of children's diet on their cognition and behavior. European journal of nutrition. 2008;47 Suppl 3:25–37. DOI: 10.1007/s00394-008-3003-x.

22. Grantham-McGregor SM, Fernald LC, Sethuraman K. Effects of Health and Nutrition on Cognitive and Behavioural Development in Children in the First Three Years of Life: Part 1: Low Birthweight, Breastfeeding, and Protein-Energy Malnutrition. Food Nutr Bull. 1999;20(1):53–75. DOI: 10.1177/156482659902000107.

23. Grantham-McGregor SM, Fernald LC, Sethuraman K. Effects of Health and Nutrition on Cognitive and Behavioural Development in Children in the First Three

Years of Life: Part 2: Infections and Micronutrient Deficiencies: Iodine, Iron, and Zinc. Food Nutr Bull. 1999;20(1):76–99. DOI: 10.1177/156482659902000108.

24. Morley R. Nutrition and cognitive development. Nutrition (Burbank, Los Angeles County, Calif.). 1998;14(10):752–54. DOI: 10.1016/S0899-9007(98)00076-8.

25. Sigman M, Whaley SE. The role of nutrition in the development of intelligence. In: Neisser U, editor. The rising curve: Long-term gains in IQ and related measures. Washington: American Psychological Association; 1998. p. 155–82.

26. Smollich M. Omega-3 fatty acids and brain function. Ernahrungs Umschau. 2015:170–77. DOI: 10.4455/eu.2015.032.

27. Rao TSS, Asha MR, Ramesh BN, Rao KSJ. Understanding nutrition, depression and mental illnesses. Indian journal of psychiatry. 2008;50(2):77–82. DOI: 10.4103/0019-5545.42391.

28. Melchior M, Chastang J-F, Falissard B, Galéra C, Tremblay RE, Côté SM, et al. Food insecurity and children's mental health: a prospective birth cohort study. PloS one. 2012;7(12):e52615. DOI: 10.1371/journal.pone.0052615.

29. Vander Wal JS, Mitchell ER. Psychological complications of pediatric obesity. Pediatric clinics of North America. 2011;58(6):1393-401, x. DOI: 10.1016/j.pcl.2011.09.008.

30. Canals J, Arija V, Esparó G, Murphy M, Fernández-Ballart J. Psychological problems and nutritional status in 6-year-old children. Psychological reports. 2005;96(3 Pt 1):840–42. DOI: 10.2466/pr0.96.3.840-842.

31. Brands B, Egan B, Györei E, López-Robles JC, Gage H, Campoy C, et al. A qualitative interview study on effects of diet on children's mental state and performance. Evaluation of perceptions, attitudes and beliefs of parents in four European countries. Appetite. 2012;58(2):739–46. DOI: 10.1016/j.appet.2012.01.004.

32. Kudrevatykh MA, Shatkhanova NA. Assessment of School Nutrition and Its Impact on Physical Development and Morbidity. Acta biomedica scientifica. 2020;5(5):81–85. DOI: 10.29413/abs.2020-5.5.11.

33. Green Corkins K, Teague EE. Pediatric Nutrition Assessment. Nut in Clin Prac. 2017;32(1):40–51. DOI: 10.1177/0884533616679639.

34. Milani GP, Silano M, Mazzocchi A, Bettocchi S, Cosmi V de, Agostoni C. Personalized nutrition approach in pediatrics: a narrative review. Pediatric research. 2021;89(2):384–88. DOI: 10.1038/s41390-020-01291-8.

35. Shvabskaia OB, Karamnova NS, Izmailova OV. Healthy Diet: New Rations for Individual Use. Racional'naâ farmakoterapiâ v kardiologii. 2020;16(6):958–65. DOI: 10.20996/1819-6446-2020-12-12.

36. Herforth A, Arimond M, Álvarez-Sánchez C, Coates J, Christianson K, Muehlhoff E. A Global Review of Food-Based Dietary Guidelines. Advances in nutrition (Bethesda, Md.). 2019;10(4):590–605. DOI: 10.1093/advances/nmy130.

37. Willett WC, Stampfer MJ. Current evidence on healthy eating. Annual review of public health. 2013;34:77–95. DOI: 10.1146/annurev-publhealth-031811-124646.

38. Lesser LI, Mazza MC, Lucan SC. Nutrition myths and healthy dietary advice in clinical practice. American family physician. 2015;91(9):634–38.

39. Booth DA. Nutrients epidemiology or healthy dietary practices? Appetite. 2002;38(1):69-70; discussion 87-8. DOI: 10.1006/appe.2001.0447.

40. Tucker KL. Eat a variety of healthful foods: old advice with new support. Nutrition reviews. 2001;59(5):156–58. DOI: 10.1111/J.1753-4887.2001.TB07005.X.

41. Truswell AS. Practical and realistic approaches to healthier diet modifications. The American journal of clinical nutrition. 1998;67(3 Suppl):583S-90S. DOI: 10.1093/ajcn/67.3.583S.

42. Taras HL, Nader PR, Sallis JF, Patterson TL, Rupp JW. Early childhood diet: Recommendations of pediatric health care providers. Journal of the American Dietetic Association. 1988;88(11):1417–21. DOI: 10.1016/s0002-8223%2821%2908027-5.

43. Baroni L, Goggi S, Battaglino R, Berveglieri M, Fasan I, Filippin D, et al. Vegan Nutrition for Mothers and Children: Practical Tools for Healthcare Providers. Nutrients. 2018;11(1). DOI: 10.3390/nu11010005.

44. Schürmann S, Kersting M, Alexy U. Vegetarian diets in children: a systematic review. European journal of nutrition. 2017;56(5):1797–817. DOI: 10.1007/s00394-017-1416-0.

45. Kalhoff H, Lücke T, Kersting M. Praktische Beratung und Betreuung bei vegetarischer Kinderernährung. Monatsschr Kinderheilkd. 2019;167(9):803–12. DOI: 10.1007/s00112-019-0730-4.

46. Alexy U, Weder S, Hoffmann M, Keller M. Vegane Kinderernährung: Hinweise zur praktischen Umsetzung. Aktuel Ernahrungsmed. 2020;45(02):93–103. DOI: 10.1055/a-1066-4342.

47. Richter M, Boeing H, Deutsche Gesellschaft für Ernährungs e.V. (DGE). Vegan Diet. Position of the German Nutrition Society (DGE). Ernahrungs Umschau. 2016:92–102. DOI: 10.4455/eu.2016.021.

48. Daly AN, O'Sullivan EJ, Kearney JM. Considerations for health and food choice in adolescents. The Proceedings of the Nutrition Society. 2022;81(1):75–86. DOI: 10.1017/S0029665121003827.

49. Fox EL, Timmer A. Children's and adolescents' characteristics and interactions with the food system. Global Food Security. 2020;27:100419. DOI: 10.1016/j.gfs.2020.100419.

50. Nemec K. Cultural Awareness of Eating Patterns in the Health Care Setting. Clinical liver disease. 2020;16(5):204–07. DOI: 10.1002/cld.1019.

51. Pilgrim K, Bohnet-Joschko S. Selling health and happiness how influencers communicate on Instagram about dieting and exercise: mixed methods research. BMC public health. 2019;19(1):1054. DOI: 10.1186/s12889-019-7387-8.

52. Klassen KM, Douglass CH, Brennan L, Truby H, Lim MSC. Social media use for nutrition outcomes in young adults: a mixed-methods systematic review. The international journal of behavioral nutrition and physical activity. 2018;15(1):70. DOI: 10.1186/s12966-018-0696-y.

53. Coates AE, Hardman CA, Halford JCG, Christiansen P, Boyland EJ. Social Media Influencer Marketing and Children's Food Intake: A Randomized Trial. Pediatrics. 2019;143(4). DOI: 10.1542/peds.2018-2554.

54. Alruwaily A, Mangold C, Greene T, Arshonsky J, Cassidy O, Pomeranz JL, et al. Child Social Media Influencers and Unhealthy Food Product Placement. Pediatrics. 2020;146(5). DOI: 10.1542/peds.2019-4057.

55. Mettenheim W von, Wiedmann K-P. Social Influencers and Healthy Nutrition – The Challenge of Overshadowing Effects and Uninvolved Consumers. Journal of Food Products Marketing. 2021;27(8-9):365–83. DOI: 10.1080/10454446.2022.2028692.

56. Ricciardelli LA, McCabe MP. Children's body image concerns and eating disturbance: a review of the literature. Clinical psychology review. 2001;21(3):325–44. DOI: 10.1016/S0272-7358%2899%2900051-3.

57. Sadeghirad B, Duhaney T, Motaghipisheh S, Campbell NRC, Johnston BC. Influence of unhealthy food and beverage marketing on children's dietary intake and preference: a systematic review and meta-analysis of randomized trials. Obesity reviews : an official journal of the International Association for the Study of Obesity. 2016;17(10):945–59. DOI: 10.1111/obr.12445.

58. Rogers A, Wilkinson S, Downie O, Truby H. Communication of nutrition information by influencers on social media: A scoping review. Health promotion journal of Australia : official journal of Australian Association of Health Promotion Professionals. 2022;33(3):657–76. DOI: 10.1002/hpja.563.

59. Reese I, Schäfer C, Ballmer-Weber B, Beyer K, Dölle-Bierke S, van Dullemen S, et al. Vegane Kostformen aus allergologischer Sicht. Positionspapier der Arbeitsgruppe Nahrungsmittelallergie der DGAKI. AL. 2023;46(04):225–54. DOI: 10.5414/ALX02400.

60. Dumas A, Robitaille J, Jette SL. Lifestyle as a choice of necessity: Young women, health and obesity. Soc Theory Health. 2014;12(2):138–58. DOI: 10.1057/STH.2013.25.

61. Penagini F, Dilillo D, Meneghin F, Mameli C, Fabiano V, Zuccotti GV. Gluten-free diet in children: an approach to a nutritionally adequate and balanced diet. Nutrients. 2013;5(11):4553–65. DOI: 10.3390/nu5114553.

62. Vici G, Belli L, Biondi M, Polzonetti V. Gluten free diet and nutrient deficiencies: A review. Clinical nutrition (Edinburgh, Scotland). 2016;35(6):1236–41. DOI: 10.1016/j.clnu.2016.05.002.

63. Reber E, Gomes F, Vasiloglou MF, Schuetz P, Stanga Z. Nutritional Risk Screening and Assessment. Journal of clinical medicine. 2019;8(7). DOI: 10.3390/jcm8071065.

64. Harnack L, Block G, Lane S. Influence of Selected Environmental and Personal Factors on Dietary Behavior for Chronic Disease Prevention: A Review of the Literature. Journal of Nutrition Education. 1997;29(6):306–12. DOI: 10.1016/S0022-3182%2897%2970244-9.

65. Ferrie S. What is nutritional assessment? A quick guide for critical care clinicians. Australian critical care : official journal of the Confederation of Australian Critical Care Nurses. 2020;33(3):295–99. DOI: 10.1016/j.aucc.2020.02.005.

66. Huang JS, Donohue M, Golnari G, Fernandez S, Walker-Gallego E, Galvan K, et al. Pediatricians' weight assessment and obesity management practices. BMC pediatrics. 2009;9:19. DOI: 10.1186/1471-2431-9-19.

67. Braet C, O'Malley G, Weghuber D, Vania A, Erhardt E, Nowicka P, et al. The assessment of eating behaviour in children who are obese: a psychological approach. A position paper from the European childhood obesity group. Obesity facts. 2014;7(3):153–64. DOI: 10.1159/000362391.

68. Mascarenhas MR, Zemel B, Stallings VA. Nutritional assessment in pediatrics. Nutrition (Burbank, Los Angeles County, Calif.). 1998;14(1):105–15. DOI: 10.1016/S0899-9007%2897%2900226-8.

69. https://www.associationfornutrition.org/wp-content/uploads/2021/10/2021-UK-Undergraduate-Curriculum-in-Nutrition-for-Medical-Doctors-FINAL.pdf (accessed January 23, 2025).

70. Ahlers-Schmidt CR, Kroeker D, Chesser A, Hart T, Brannon J. Visual Recognition of Child Body Mass Index by Medical Students, Resident Physicians, and Community Physicians. kjm. 2010;3(5):7–14. DOI: 10.17161/KJM.V3I5.11323.

71. Spurrier NJ, Magarey A, Wong C. Recognition and management of childhood overweight and obesity by clinicians. Journal of paediatrics and child health. 2006;42(7-8):411–18. DOI: 10.1111/j.1440-1754.2006.00890.x.

72. Jeong Eun, Jung Mi-Ra. 청소년의 건강정보이해능력, 체형인식과 건강관련위험인식이 건강증진행위에 미치는 요인. Journal of Convergence for Information Technology. 2020;10(7):49–57. DOI: 10.22156/CS4SMB.2020.10.07.049.

73. Torun B. Energy requirements of children and adolescents. Public health nutrition. 2005;8(7A):968–93. DOI: 10.1079/PHN2005791.

74. Woodruff SJ, Hanning RM, Barr SI. Energy recommendations for normal weight, overweight and obese children and adolescents: are different equations necessary? Obesity reviews : an official journal of the International Association for the Study of Obesity. 2009;10(1):103–08. DOI: 10.1111/j.1467-789X.2008.00525.x.

75. Büning-Fesel M. Das Bundeszentrum für Ernährung (BZfE) – neuer Akteur für Ernährungsfragen. Ernährung & Medizin. 2017;32(04):190–92. DOI: 10.1055/s-0043-119855.

76. Small L, Lane H, Vaughan L, Melnyk B, McBurnett D. A systematic review of the evidence: the effects of portion size manipulation with children and portion education/training interventions on dietary intake with adults. Worldviews on evidence-based nursing. 2013;10(2):69–81. DOI: 10.1111/j.1741-6787.2012.00257.x.

77. More JA, Emmett PM. Evidenced-based, practical food portion sizes for preschool children and how they fit into a well balanced, nutritionally adequate diet. Journal of human nutrition and dietetics : the official journal of the British Dietetic Association. 2015;28(2):135–54. DOI: 10.1111/jhn.12228.

78. Birch LL, Savage JS, Fisher JO. Right sizing prevention. Food portion size effects on children's eating and weight. Appetite. 2015;88:11–16. DOI: 10.1016/j.appet.2014.11.021.

79. https://www.ernaehrungs-umschau.de/fileadmin/Ernaehrungs-Umschau/pdfs/pdf_2013/11_13/EU11_2013_M644_M645.qxd.pdf (accessed January 23, 2025).

80. Gidding SS, Dennison BA, Birch LL, Daniels SR, Gillman MW, Lichtenstein AH, et al. Dietary recommendations for children and adolescents: a guide for practitioners: consensus statement from the American Heart Association. Circulation. 2005;112(13):2061–75. DOI: 10.1161/CIRCULATIONAHA.105.169251.

81. Bristow KS, Mwatsama M, Capewell S, Lloyd-Williams F. P39 Healthy eating for children in early years settings: a systematic review of current guidance at local and national levels. Journal of Epidemiology & Community Health. 2010;64(Suppl 1):A48-A49. DOI: 10.1136/jech.2010.120477.39.

82. Harrison SE, Greenhouse D. Dietary and Nutrition Recommendations in Pediatric Primary Care: A Call to Action. Southern medical journal. 2018;111(1):12–17. DOI: 10.14423/SMJ.0000000000000754.

83. Hondt L de, Gorsen SL, Verburgh P, Paepe K de, Muyldermans J, Tommelein E. Health Care Providers' Perspective and Knowledge about Peri-Surgical Medication and Practices in Breastfeeding Women. International journal of environmental research and public health. 2023;20(4). DOI: 10.3390/ijerph20043379.

84. Sesselberg TS, Klein JD, O'Connor KG, Johnson MS. Screening and counseling for childhood obesity: results from a national survey. Journal of the American Board of Family Medicine : JABFM. 2010;23(3):334–42. DOI: 10.3122/jabfm.2010.03.090070.

85. Dash S, Delibasic V, Alsaeed S, Ward M, Jefferson K, Manca DP, et al. Knowledge, Attitudes and Behaviours Related to Physician-Delivered Dietary Advice for Patients with Hypertension. Journal of community health. 2020;45(5):1067–72. DOI: 10.1007/s10900-020-00831-x.

86. Harillo-Acevedo D, Ramos-Morcillo AJ, Ruzafa-Martinez M. Factors associated with breastfeeding support from health care professionals by implementing a Clinical Practice Guideline. Birth (Berkeley, Calif.). 2019;46(1):146–56. DOI: 10.1111/birt.12382.

87. Giglia RC, Symons M, Shaw T. The provision of alcohol and breastfeeding information by maternal health practitioners in the Australian setting. The Australian & New Zealand journal of obstetrics & gynaecology. 2019;59(2):258–64. DOI: 10.1111/ajo.12837.

88. Al-Sawalha NA, Sawalha A, Tahaineh L, Almomani B, Al-Keilani M. Healthcare providers' attitude and knowledge regarding medication use in breastfeeding women: a Jordanian national questionnaire study. Journal of obstetrics and gynaecology : the journal of the Institute of Obstetrics and Gynaecology. 2018;38(2):217–21. DOI: 10.1080/01443615.2017.1345876.

89. Lobitz CA, Yamaguchi I. Lifestyle Interventions for Elevated Blood Pressure in Childhood-Approaches and Outcomes. Current hypertension reports. 2022;24(11):589–98. DOI: 10.1007/s11906-022-01217-1.

90. Binka E, Brady TM. Real-World Strategies to Treat Hypertension Associated with Pediatric Obesity. Current hypertension reports. 2019;21(2):18. DOI: 10.1007/s11906-019-0922-2.

91. Ferreira YAM, Kravchychyn ACP, Vicente SdCF, Da Campos RMS, Tock L, Oyama LM, et al. An Interdisciplinary Weight Loss Program Improves Body Composition and Metabolic Profile in Adolescents With Obesity: Associations With the Dietary Inflammatory Index. Frontiers in nutrition. 2019;6:77. DOI: 10.3389/fnut.2019.00077.

92. Genovesi S, Orlando A, Rebora P, Giussani M, Antolini L, Nava E, et al. Effects of Lifestyle Modifications on Elevated Blood Pressure and Excess Weight in a Population of Italian Children and Adolescents. American journal of hypertension. 2018;31(10):1147–55. DOI: 10.1093/ajh/hpy096.

93. Vos RC, Huisman SD, Houdijk ECAM, Pijl H, Wit JM. The effect of family-based multidisciplinary cognitive behavioral treatment on health-related quality of life in childhood obesity. Quality of life research : an international journal of quality of life aspects of treatment, care and rehabilitation. 2012;21(9):1587–94. DOI: 10.1007/s11136-011-0079-1.

94. Recommendations for Preventive Pediatric Health Care. Pediatrics. 2000;105(3):645–46. DOI: 10.1542/peds.105.3.645.

95. Hoelscher DM, Kirk S, Ritchie L, Cunningham-Sabo L. Position of the Academy of Nutrition and Dietetics: interventions for the prevention and treatment of

pediatric overweight and obesity. Journal of the Academy of Nutrition and Dietetics. 2013;113(10):1375-94. DOI: 10.1016/j.jand.2013.08.004.

96. Silberberg M, Carter-Edwards L, Mayhew M, Murphy G, Anstrom K, Collier D, et al. Integrating Registered Dietitian Nutritionists Into Primary Care Practices to Work With Children With Overweight. American journal of lifestyle medicine. 2020;14(2):194-203. DOI: 10.1177/1559827617726950.

97. Watling RM. The positive impact of dietitians in paediatric health care. Paediatrics and Child Health. 2009;19(9):400-04. DOI: 10.1016/J.PAED.2009.05.004.

98. DerMarderosian D, Chapman HA, Tortolani C, Willis MD. Medical Considerations in Children and Adolescents with Eating Disorders. Child and adolescent psychiatric clinics of North America. 2018;27(1):1-14. DOI: 10.1016/j.chc.2017.08.002.

99. Barlow SE, Dietz WH. Management of Child and Adolescent Obesity: Summary and Recommendations Based on Reports From Pediatricians, Pediatric Nurse Practitioners, and Registered Dietitians. Pediatrics. 2002;110(Supplement_1):236-38. DOI: 10.1542/peds.110.s1.236.

100. Sastre LR, Matson S, Gruber KJ, Haldeman L. A qualitative study examining medical provider advice, barriers, and perceived effectiveness in addressing childhood obesity to patients and families from a low-income community health clinic. SAGE open medicine. 2019;7:2050312119834117. DOI: 10.1177/2050312119834117.

101. Sastre LR, van Horn LT. Family medicine physicians' report strong support, barriers and preferences for Registered Dietitian Nutritionist care in the primary care setting. Family practice. 2021;38(1):25-31. DOI: 10.1093/fampra/cmaa099.

102. Lasswell AB. Incorporating nutrition into pediatric practice: physicians and dietitians working together to improve children's health. Pediatric annals. 1992;21(10):676-7, 681-7. DOI: 10.3928/0090-4481-19921001-09.

103. Samady W, Campbell E, Aktas ON, Jiang J, Bozen A, Fierstein JL, et al. Recommendations on Complementary Food Introduction Among Pediatric Practitioners. JAMA network open. 2020;3(8):e2013070. DOI: 10.1001/jamanetworkopen.2020.13070.

104. Nguyen SP. An apple a day keeps the doctor away: children's evaluative categories of food. Appetite. 2007;48(1):114-18. DOI: 10.1016/j.appet.2006.06.001.

105. Nguyen SP. Children's Evaluative Categories and Inductive Inferences within the Domain of Food. Infant and child development. 2008;17(3):285-99. DOI: 10.1002/ICD.553.

106. Kersting M. Kinderernährung aktuell - Herausforderungen und Chancen. Ernährung & Medizin. 2013;28(01):21-24. DOI: 10.1055/s-0032-1331080.

107. Parks EP, Mascarenhas MR, Goh V. Nutrient needs and requirements during growth. In: Present Knowledge in Nutrition: Elsevier; 2020. p. 23-44.

108. Weaver CM, Dwyer J, Fulgoni VL, King JC, Leveille GA, MacDonald RS, et al. Processed foods: contributions to nutrition. The American journal of clinical nutrition. 2014;99(6):1525–42. DOI: 10.3945/ajcn.114.089284.

109. Gibson RS, Perlas L, Hotz C. Improving the bioavailability of nutrients in plant foods at the household level. The Proceedings of the Nutrition Society. 2006;65(2):160–68. DOI: 10.1079/PNS2006489.

110. Khandpur N, Neri DA, Monteiro C, Mazur A, Frelut M-L, Boyland E, et al. Ultra-Processed Food Consumption among the Paediatric Population: An Overview and Call to Action from the European Childhood Obesity Group. Annals of nutrition & metabolism. 2020;76(2):109–13. DOI: 10.1159/000507840.

111. Bailey RL, Fulgoni VL, Keast DR, Lentino CV, Dwyer JT. Do dietary supplements improve micronutrient sufficiency in children and adolescents? The Journal of pediatrics. 2012;161(5):837–42. DOI: 10.1016/j.jpeds.2012.05.009.

112. Smolinske SC. Dietary Supplements in Children. Pediatric clinics of North America. 2017;64(6):1243–55. DOI: 10.1016/j.pcl.2017.09.001.

113. Kersting M. Zusätzliche Nährstoffe bei Kindern – notwendig oder überflüssig? Ernährung & Medizin. 2016;31(01):45–48. DOI: 10.1055/s-0042-100968.

114. Appel KS, Jung C, Nowak N, Golsong N, Lindtner O. Intake of dietary supplements in infants and (young) children in Germany. Ernahrungs Umschau. 2021;68(12):224–30. DOI: 10.4455/eu.2021.048.

115. Bechthold A, Albrecht V, Leschik-Bonnet E. Beurteilung der Vitaminversorgung in Deutschland. Teil 1: Daten zur Vitaminzufuhr. Ernahrungs Umschau. 2012:324–36. DOI: 10.4455/eu.2012.974.

116. Bechthold A, Albrecht V, Leschik-Bonnet E. Beurteilung der Vitaminversorgung in Deutschland. Teil 2: Kritische Vitamine und Vitaminzufuhr in besonderen Lebenssituationen. Ernahrungs Umschau. 2012:396–401. DOI: 10.4455/eu.2012.969.

117. Delgas F, Lederer A, Holzäpfel S, Podszun MC. Supplements during lactation The more the merrier? Ernahrungs Umschau. 2024:64–75. DOI: 10.4455/eu.2023.010.

118. Picciano MF, Dwyer JT, Radimer KL, Wilson DH, Fisher KD, Thomas PR, et al. Dietary supplement use among infants, children, and adolescents in the United States, 1999-2002. Archives of pediatrics & adolescent medicine. 2007;161(10):978–85. DOI: 10.1001/ARCHPEDI.161.10.978.

119. Krebs NF. Bioavailability of dietary supplements and impact of physiologic state: infants, children and adolescents. The Journal of nutrition. 2001;131(4 Suppl):1351S-4S. DOI: 10.1093/jn/131.4.1351S.

120. Babu KM, McCormick MA, Bird SB. Pediatric Dietary Supplement Use—An Update. Clinical Pediatric Emergency Medicine. 2005;6(2):85–92. DOI: 10.1016/J.CPEM.2005.04.003.

121. Koletzko B, Brönstrup A, Cremer M, Flothkötter M, Hellmers C, Kersting M, et al. Säuglingsernährung und Ernährung der stillenden Mutter. Monatsschr Kinderheilkd. 2010;158(7):679–89. DOI: 10.1007/s00112-010-2240-2.

122. Joshi R, Kumar A, Masih S. Food hygiene practice among mothers and its association with occurrence of diarrhea in under-five children in selected rural community area. Int J Med Sci Public Health. 2020(0):1. DOI: 10.5455/ijmsph.2020.1233929122019.

123. Bundesinstitut für Risikobewertung. Empfehlungen zur hygienischen Zubereitung pulverförmiger Säuglingsnahrung: Aktualisierte Stellungnahme Nr. 009/2022 des BfR vom 29. März 2022: Bundesbehörden und Einrichtungen im Geschäftsbereich des Bundesministeriums für Ernährung und Landwirtschaft (BMEL); 2022.

124. Kuchma VR. Gigienicheskoe i ékologicheskoe obuchenie vracheĭ-pediatrov. Gigiena i sanitariia. 1999(4):30–33.

125. Nawarathne LC, Wanniarachchi PC, Peiris HS, Abeysundara PDA. Assessment of Food Safety Knowledge of Food Handlers and the Level of Implementation of Good Manufacturing Practices at Restaurants in Kegalle District, Sri Lanka. VJScience. 2022;25(02). DOI: 10.31357/vjs.v25i02.6175.

126. Kendall P, Medeiros LC, Hillers V, Chen G, DiMascola S. Food handling behaviors of special importance for pregnant women, infants and young children, the elderly, and immune-compromised people. Journal of the American Dietetic Association. 2003;103(12):1646–49. DOI: 10.1016/J.JADA.2003.09.027.

127. Sockett PN, Rodgers FG. Enteric and foodborne disease in children: A review of the influence of food- and environment-related risk factors. Paediatrics & child health. 2001;6(4):203–09. DOI: 10.1093/PCH/6.4.203.

128. Marcus R. New information about pediatric foodborne infections: the view from FoodNet. Current opinion in pediatrics. 2008;20(1):79–84. DOI: 10.1097/MOP.0b013e3282f43067.

129. Trepka MJ, Newman FL, Dixon Z, Huffman FG. Food safety practices among pregnant women and mothers in the women, infants, and children program, Miami, Florida. Journal of food protection. 2007;70(5):1230–37. DOI: 10.4315/0362-028X-70.5.1230.

130. Bazaco MC, Albrecht SA, Malek AM. Preventing foodborne infection in pregnant women and infants. Nursing for women's health. 2008;12(1):46–55. DOI: 10.1111/j.1751-486X.2007.00275.x.

131. Campbell AK, Matthews SB, Vassel N, Cox CD, Naseem R, Chaichi J, et al. Bacterial metabolic 'toxins': a new mechanism for lactose and food intolerance, and irritable bowel syndrome. Toxicology. 2010;278(3):268–76. DOI: 10.1016/j.tox.2010.09.001.

132. Whipps J, Mort SC, Beverly EA, Guseman EH. Influence of Osteopathic Medical Students' Personal Health on Attitudes Toward Counseling Obese Pediatric Patients. The Journal of the American Osteopathic Association. 2019;119(8):488–98. DOI: 10.7556/jaoa.2019.090.

133. Amoore BY, Gaa PK, Amalba A, Mogre V. Nutrition education intervention improves medical students' dietary habits and their competency and self-efficacy in providing nutrition care: A pre, post and follow-up quasi-experimental study. Frontiers in nutrition. 2023;10:1063316. DOI: 10.3389/fnut.2023.1063316.

134. Lavelle F, Spence M, Hollywood L, McGowan L, Surgenor D, McCloat A, et al. Learning cooking skills at different ages: a cross-sectional study. The international journal of behavioral nutrition and physical activity. 2016;13(1):119. DOI: 10.1186/s12966-016-0446-y.

135. Nelson SA, Corbin MA, Nickols-Richardson SM. A call for culinary skills education in childhood obesity-prevention interventions: current status and peer influences. Journal of the Academy of Nutrition and Dietetics. 2013;113(8):1031–36. DOI: 10.1016/j.jand.2013.05.002.

136. Allirot X, Da Quinta N, Chokupermal K, Urdaneta E. Involving children in cooking activities: A potential strategy for directing food choices toward novel foods containing vegetables. Appetite. 2016;103:275–85. DOI: 10.1016/j.appet.2016.04.031.

137. Kim SA, Grimm KA, May AL, Harris DM, Kimmons J, Foltz JL. Strategies for pediatric practitioners to increase fruit and vegetable consumption in children. Pediatric clinics of North America. 2011;58(6):1439-53, xi. DOI: 10.1016/j.pcl.2011.09.011.

138. Dean M, O'Kane C, Issartel J, McCloat A, Mooney E, Gaul D, et al. Guidelines for designing age-appropriate cooking interventions for children: The development of evidence-based cooking skill recommendations for children, using a multidisciplinary approach. Appetite. 2021;161:105125. DOI: 10.1016/j.appet.2021.105125.

139. Ogata BN, Hayes D. Position of the Academy of Nutrition and Dietetics: nutrition guidance for healthy children ages 2 to 11 years. Journal of the Academy of Nutrition and Dietetics. 2014;114(8):1257–76. DOI: 10.1016/j.jand.2014.06.001.

140. Günther ALB, Buyken AE, Kroke A. The influence of habitual protein intake in early childhood on BMI and age at adiposity rebound: results from the DONALD Study. International journal of obesity (2005). 2006;30(7):1072–79. DOI: 10.1038/sj.ijo.0803288.

141. Arnesen EK, Thorisdottir B, Lamberg-Allardt C, Bärebring L, Nwaru B, Dierkes J, et al. Protein intake in children and growth and risk of overweight or obesity: A systematic review and meta-analysis. Food & nutrition research. 2022;66. DOI: 10.29219/fnr.v66.8242.

142. Weber M, Grote V, Closa-Monasterolo R, Escribano J, Langhendries J-P, Dain E, et al. Lower protein content in infant formula reduces BMI and obesity risk at school age: follow-up of a randomized trial. The American journal of clinical nutrition. 2014;99(5):1041–51. DOI: 10.3945/ajcn.113.064071.

143. Braun KV, Erler NS, Kiefte-de Jong JC, Jaddoe VW, van den Hooven EH, Franco OH, et al. Dietary Intake of Protein in Early Childhood Is Associated with Growth Trajectories between 1 and 9 Years of Age. The Journal of nutrition. 2016;146(11):2361–67. DOI: 10.3945/JN.116.237164.

144. Hudson JL, Baum JI, Diaz EC, Børsheim E. Dietary Protein Requirements in Children: Methods for Consideration. Nutrients. 2021;13(5). DOI: 10.3390/nu13051554.

145. Elango R, Humayun MA, Ball RO, Pencharz PB. Protein requirement of healthy school-age children determined by the indicator amino acid oxidation method. The American journal of clinical nutrition. 2011;94(6):1545–52. DOI: 10.3945/ajcn.111.012815.

146. Koletzko B, Kries R von, Closa R, Escribano J, Scaglioni S, Giovannini M, et al. Lower protein in infant formula is associated with lower weight up to age 2 y: a randomized clinical trial. The American journal of clinical nutrition. 2009;89(6):1836–45. DOI: 10.3945/ajcn.2008.27091.

147. Koletzko B, Demmelmair H, Grote V, Prell C, Weber M. High protein intake in young children and increased weight gain and obesity risk. The American journal of clinical nutrition. 2016;103(2):303–04. DOI: 10.3945/ajcn.115.128009.

148. Dewey K. Meeting protein needs at 6 to 24 months of age. Food Nutr Bull. 2013;34(2):240–41. DOI: 10.1177/156482651303400216.

149. Volterman KA, Atkinson SA. Protein Needs of Physically Active Children. Pediatric exercise science. 2016;28(2):187–93. DOI: 10.1123/pes.2015-0257.

150. Embleton ND, Cooke RJ. Protein requirements in preterm infants: effect of different levels of protein intake on growth and body composition. Pediatric research. 2005;58(5):855–60. DOI: 10.1203/01.PDR.0000182586.46532.7C.

151. Hörnell A, Lagström H, Lande B, Thorsdottir I. Protein intake from 0 to 18 years of age and its relation to health: a systematic literature review for the 5th Nordic Nutrition Recommendations. Food & nutrition research. 2013;57. DOI: 10.3402/fnr.v57i0.21083.

152. Kersting M, Schöch G. Normale Ernährung von Neugeborenen, Säuglingen, Kindern und Jugendlichen. In: Lentze MJ, Schulte FJ, Schaub J, Spranger J, editors. Pädiatrie. Berlin, Heidelberg: Springer Berlin Heidelberg; 2007. p. 181–207.

153. Protein and amino acid requirements in human nutrition: Report of a joint WHO/FAO/UNU Expert Consultation ; [Geneva, 9 - 16 April 2002. WHO technical report series, Vol 935. Geneva: WHO; 2007.

154. Schwingshackl L, Zähringer J, Beyerbach J, Werner SS, Nagavci B, Heseker H, et al. A Scoping Review of Current Guidelines on Dietary Fat and Fat Quality. Annals of nutrition & metabolism. 2021;77(2):65–82. DOI: 10.1159/000515671.

155. Koletzko B, Dokoupil K, Reitmayr S, Weimert-Harendza B, Keller E. Dietary fat intakes in infants and primary school children in Germany. The American journal of clinical nutrition. 2000;72(5 Suppl):1392S-1398S. DOI: 10.1093/ajcn/72.5.1392s.

156. Butte NF. Fat intake of children in relation to energy requirements. The American journal of clinical nutrition. 2000;72(5 Suppl):1246S-1252S. DOI: 10.1093/ajcn/72.5.1246s.

157. Lewin GA, Schachter HM, Yuen D, Merchant P, Mamaladze V, Tsertsvadze A. Effects of omega-3 fatty acids on child and maternal health. Evidence report/technology assessment (Summary). 2005(118):1–11.

158. Sibbons C, Boyle L, Burdge GC, Umpleby M, Lilycrop KA, Hartwick CA, et al. Evaluation of fatty acid status in children of different nationalities. Proc. Nutr. Soc. 2015;74(OCE1). DOI: 10.1017/S0029665115001093.

159. Nevins JEH, Donovan SM, Snetselaar L, Dewey KG, Novotny R, Stang J, et al. Omega-3 Fatty Acid Dietary Supplements Consumed During Pregnancy and Lactation and Child Neurodevelopment: A Systematic Review. The Journal of nutrition. 2021;151(11):3483–94. DOI: 10.1093/jn/nxab238.

160. Shulkin ML, Pimpin L, Bellinger D, Kranz S, Duggan C, Fawzi W, et al. Effects of omega-3 supplementation during pregnancy and youth on neurodevelopment and cognition in childhood: a systematic review and meta-analysis. The FASEB Journal. 2016;30(S1). DOI: 10.1096/FASEBJ.30.1_SUPPLEMENT.295.5.

161. Ryan AS, Astwood JD, Gautier S, Kuratko CN, Nelson EB, Salem N. Effects of long-chain polyunsaturated fatty acid supplementation on neurodevelopment in childhood: a review of human studies. Prostaglandins, leukotrienes, and essential fatty acids. 2010;82(4-6):305–14. DOI: 10.1016/j.plefa.2010.02.007.

162. Ciccone MM, Scicchitano P, Gesualdo M, Zito A, Carbonara S, Ricci G, et al. The role of omega-3 polyunsaturated fatty acids supplementation in childhood: a review. Recent patents on cardiovascular drug discovery. 2013;8(1):42–55. DOI: 10.2174/1574890111308010006.

163. Uusitalo L, Nevalainen J, Salminen I, Ovaskainen M-L, Kronberg-Kippilä C, Ahonen S, et al. Fatty acids in serum and diet--a canonical correlation analysis among toddlers. Maternal & child nutrition. 2013;9(3):381–95. DOI: 10.1111/j.1740-8709.2011.00374.x.

164. Monnard C, Fleith M. Total Fat and Fatty Acid Intake among 1-7-Year-Old Children from 33 Countries: Comparison with International Recommendations. Nutrients. 2021;13(10). DOI: 10.3390/nu13103547.

165. Harika RK, Cosgrove MC, Osendarp SJM, Verhoef P, Zock PL. Fatty acid intakes of children and adolescents are not in line with the dietary intake recommendations for future cardiovascular health: a systematic review of dietary intake data from thirty countries. The British journal of nutrition. 2011;106(3):307–16. DOI: 10.1017/S0007114511001528.

166. Cerdó T, Diéguez E, Campoy C. Infant growth, neurodevelopment and gut microbiota during infancy: which nutrients are crucial? Current opinion in clinical nutrition and metabolic care. 2019;22(6):434–41. DOI: 10.1097/MCO.0000000000000606.

167. Edwin Thanarajah S, DiFeliceantonio AG, Albus K, Kuzmanovic B, Rigoux L, Iglesias S, et al. Habitual daily intake of a sweet and fatty snack modulates reward processing in humans. Cell metabolism. 2023;35(4):571-584.e6. DOI: 10.1016/j.cmet.2023.02.015.

168. Wolfram G, Bechthold A, Boeing H, Ellinger S, Hauner H, Kroke A, et al. Evidence-Based Guideline of the German Nutrition Society: Fat Intake and Prevention of Selected Nutrition-Related Diseases. Annals of nutrition & metabolism. 2015;67(3):141–204. DOI: 10.1159/000437243.

169. https://www.dge.de/fileadmin/Bilder/wissenschaft/referenzwerte/energie/DGE-Positionspapier-Richtwerte-Energiezufuhr-KH-und-Fett.pdf (accessed January 30, 2025).

170. Kranz S, Brauchla M, Slavin JL, Miller KB. What do we know about dietary fiber intake in children and health? The effects of fiber intake on constipation, obesity, and diabetes in children. Advances in nutrition (Bethesda, Md.). 2012;3(1):47–53. DOI: 10.3945/an.111.001362.

171. Hojsak I, Benninga MA, Hauser B, Kansu A, Kelly VB, Stephen AM, et al. Benefits of dietary fibre for children in health and disease. Archives of disease in childhood. 2022;107(11):973–79. DOI: 10.1136/archdischild-2021-323571.

172. Fidler Mis N, Braegger C, Bronsky J, Campoy C, Domellöf M, Embleton ND, et al. Sugar in Infants, Children and Adolescents: A Position Paper of the European Society for Paediatric Gastroenterology, Hepatology and Nutrition Committee on Nutrition. Journal of pediatric gastroenterology and nutrition. 2017;65(6):681–96. DOI: 10.1097/MPG.0000000000001733.

173. Sunardi D. Strategy to fulfill fiber requirement in children. World Nutr J. 2022;5(S3):8–9. DOI: 10.25220/wnj.v05.s3.0005.

174. Boeing H, Bechthold A, Bub A, Ellinger S, Haller D, Kroke A, et al. Critical review: vegetables and fruit in the prevention of chronic diseases. European journal of nutrition. 2012;51(6):637–63. DOI: 10.1007/s00394-012-0380-y.

175. Koletzko B, Bührer C, Ensenauer R, Jochum F, Kahlhoff H, Lawrenz B, et al. Beikostprodukte aus Quetschbeuteln. Monatsschr Kinderheilkd. 2019;167(6):539–44. DOI: 10.1007/s00112-019-0670-z.

176. Hauner H, Bechthold A, Boeing H, Brönstrup A, Buyken A, Leschik-Bonnet E, et al. Evidence-based guideline of the German Nutrition Society: carbohydrate intake and prevention of nutrition-related diseases. Annals of nutrition & metabolism. 2012;60 Suppl 1:1–58. DOI: 10.1159/000335326.

177. Ernst JB, Arens-Azevedo U, Bosy-Westphal A, Zwaan M de, Egert S. Quantitative recommendation on sugar intake in Germany. Short version of the consensus paper by the German Obesity Society (DAG), German Diabetes Society (DDG) and German Nutrition Society (DGE). Ernahrungs Umschau. 2019:26–34. DOI: 10.4455/eu.2019.006.

178. Hojsak I, Chourdakis M, Gerasimidis K, Hulst J, Huysentruyt K, Moreno-Villares JM, et al. What are the new guidelines and position papers in pediatric nutrition: A 2015-2020 overview. Clinical nutrition ESPEN. 2021;43:49–63. DOI: 10.1016/j.clnesp.2021.03.004.

179. Farag MA, Hamouda S, Gomaa S, Agboluaje AA, Hariri MLM, Yousof SM. Dietary Micronutrients from Zygote to Senility: Updated Review of Minerals' Role and Orchestration in Human Nutrition throughout Life Cycle with Sex Differences. Nutrients. 2021;13(11). DOI: 10.3390/nu13113740.

180. Binns C, Lee MK, Kagawa M. Nutrients in Infancy: Progress and Prospects. Nutrients. 2017;9(10). DOI: 10.3390/nu9101131.

181. Georgieff MK. Nutrition and the developing brain: nutrient priorities and measurement. The American journal of clinical nutrition. 2007;85(2):614S-620S. DOI: 10.1093/ajcn/85.2.614S.

182. Ilich JZ, Brownbill RA. Nutrition Through the Life Span: Needs and Health Concerns in Critical Periods. In: Miller TW, editor. Handbook of Stressful Transitions Across the Lifespan. New York, NY: Springer New York; 2010. p. 625–41.

183. Jiménez Ortega AI, Martínez García RM, Velasco Rodríguez-Belvis M, Ruiz Herrero J. De lactante a niño. Alimentación en diferentes etapas. Nutricion hospitalaria. 2017;34(Suppl 4):3–7. DOI: 10.20960/nh.1563.

184. Ekweagwu E, Ekwe Agwu A, Madukwe E. The role of micronutrients in child health: A review of the literature. African Journal of Biotechnology. 2008;7.

185. Borrmann A, Mensink GBM. Obst- und Gemüsekonsum von Kindern und Jugendlichen in Deutschland : Ergebnisse der KiGGS-Welle 1. Bundesgesundheitsblatt, Gesundheitsforschung, Gesundheitsschutz. 2015;58(9):1005–14. DOI: 10.1007/s00103-015-2208-4.

186. Ramsay SA, Eskelsen AK, Branen LJ, Armstrong Shultz J, Plumb J. Nutrient Intake and Consumption of Fruit and Vegetables in Young Children. ICAN: Infant, Child, & Adolescent Nutrition. 2014;6(6):332–44. DOI: 10.1177/1941406414549622.

187. Spalinger J. Vitamine bei Kindern – Fakten und Kontroversen. Paediatr. Paedolog. Austria. 2017;52(4):151–55. DOI: 10.1007/s00608-017-0491-z.

188. Safronova AI, Pyr'eva EA, Georgieva OV. Beverages in child nutrition. Russian Journal of Woman and Child Health. 2022;5(1):78–84. DOI: 10.32364/2618-8430-2022-5-1-78-84.

189. Fulgoni VL, Quann EE. National trends in beverage consumption in children from birth to 5 years: analysis of NHANES across three decades. Nutrition journal. 2012;11:92. DOI: 10.1186/1475-2891-11-92.

190. Bleich SN, Vercammen KA, Koma JW, Li Z. Trends in Beverage Consumption Among Children and Adults, 2003-2014. Obesity (Silver Spring, Md.). 2018;26(2):432–41. DOI: 10.1002/oby.22056.

191. Patel AI, Cabana MD. Encouraging healthy beverage intake in child care and school settings. Current opinion in pediatrics. 2010;22(6):779–84. DOI: 10.1097/MOP.0b013e32833f2fe2.

192. Rampersaud GC, Bailey LB, Kauwell GPA. National survey beverage consumption data for children and adolescents indicate the need to encourage a shift toward more nutritive beverages. Journal of the American Dietetic Association. 2003;103(1):97–100. DOI: 10.1053/JADA.2003.50006.

193. Grummon AH, Sokol RL, Hecht CA, Patel AI. Measuring beverage consumption in US children and adolescents: a systematic review. Obesity reviews : an official journal of the International Association for the Study of Obesity. 2018;19(8):1017–27. DOI: 10.1111/obr.12692.

194. Seifert SM, Schaechter JL, Hershorin ER, Lipshultz SE. Health effects of energy drinks on children, adolescents, and young adults. Pediatrics. 2011;127(3):511–28. DOI: 10.1542/peds.2009-3592.

195. Kalhoff H, Hilbig A, Libuda L. Trinken – was und wie viel? Kinder- und Jugendmedizin. 2015;15(01):7–12. DOI: 10.1055/s-0038-1629248.

196. Muth ND. Pediatricians Play Important Role in Decreasing Sugary Drink Intake in Young Children. NAM Perspectives. 2018;8(3). DOI: 10.31478/201803A.

197. D'Anci KE, Constant F, Rosenberg IH. Hydration and Cognitive Function in Children. Nutrition reviews. 2006;64(10):457–64. DOI: 10.1111/J.1753-4887.2006.TB00176.X.

198. Committee on Nutrition and the Council on Sports Medicine and Fitness. Sports drinks and energy drinks for children and adolescents: are they appropriate? Pediatrics. 2011;127(6):1182–89. DOI: 10.1542/peds.2011-0965.

199. Lessen R, Kavanagh K. Position of the academy of nutrition and dietetics: promoting and supporting breastfeeding. Journal of the Academy of Nutrition and Dietetics. 2015;115(3):444–49. DOI: 10.1016/j.jand.2014.12.014.

200. Reiss K. Akzeptanz der Handlungsempfehlungen zur Säuglingsernährung und Ernährung in der Schwangerschaft des Netzwerks „Gesund ins Leben" – Konsequenzen für die Aus- und Weiterbildung von Hebammen?: German Medical Science GMS Publishing House; 2016.

201. Bührer C, Genzel-Boroviczény O, Jochum F, Kauth T, Kersting M, Koletzko B, et al. Ernährung gesunder Säuglinge. Monatsschr Kinderheilkd. 2014;162(6):527–38. DOI: 10.1007/s00112-014-3129-2.

202. Kramer MS, Kakuma R. Optimal duration of exclusive breastfeeding. The Cochrane database of systematic reviews. 2012;2012(8):CD003517. DOI: 10.1002/14651858.CD003517.pub2.

203. Ley D, Beghin L, Morcel J, Flamein F, Garabedian C, Accart B, et al. Impact of early life nutrition on gut health in children: a prospective clinical study. BMJ open. 2021;11(9):e050432. DOI: 10.1136/bmjopen-2021-050432.

204. Kommerzielle Muttermilchverstärker aus humaner Milch: unzureichend belegter Nutzen und hohe Kosten. Monatsschr Kinderheilkd. 2019;167(2):145–48. DOI: 10.1007/s00112-018-0503-5.

205. Breastfeeding and the use of human milk. Pediatrics. 2012;129(3):e827-41. DOI: 10.1542/peds.2011-3552.

206. Aumeistere L, Ciproviča I, Zavadska D, Andersons J, Volkovs V, Ceļmalniece K. Impact of Maternal Diet on Human Milk Composition Among Lactating Women in Latvia. Medicina (Kaunas, Lithuania). 2019;55(5). DOI: 10.3390/medicina55050173.

207. Achón M, Úbeda N, García-González Á, Partearroyo T, Varela-Moreiras G. Effects of Milk and Dairy Product Consumption on Pregnancy and Lactation Outcomes: A Systematic Review. Advances in nutrition (Bethesda, Md.). 2019;10(suppl_2):S74-S87. DOI: 10.1093/advances/nmz009.

208. O'Connor DL, Houghton LA, Sherwood KL. Nutrition Issues During Lactation. In: Lammi-Keefe CJ, Couch SC, Philipson EH, editors. Handbook of Nutrition and Pregnancy. Nutrition and Health. Totowa, NJ: Humana Press; 2008. p. 257–82.

209. Fulhan J, Collier S, Duggan C. Update on pediatric nutrition: breastfeeding, infant nutrition, and growth. Current opinion in pediatrics. 2003;15(3):323–32. DOI: 10.1097/00008480-200306000-00017.

210. Koletzko B, Bauer CP, Bung P, Cremer M, Flothkötter M, Hellmers C, et al. Ernährung in Schwangerschaft, Stillzeit und im ersten Lebensjahr - Handlungsempfehlungen des Netzwerks „Gesund ins Leben" als einheitliche Basis für die Beratung (werdender) Eltern. Aktuel Ernahrungsmed. 2012;37(03). DOI: 10.1055/s-0032-1312494.

211. Watt J, Mead J. What paediatricians need to know about breastfeeding. Paediatrics and Child Health. 2013;23(8):362–66. DOI: 10.1016/J.PAED.2013.05.004.

212. Picariello G, Cicco M de, Nocerino R, Paparo L, Mamone G, Addeo F, et al. Excretion of Dietary Cow's Milk Derived Peptides Into Breast Milk. Frontiers in nutrition. 2019;6:25. DOI: 10.3389/fnut.2019.00025.

213. Kalhoff H, Kersting M. Pädiatrische Ernährung. Eisenreiche Beikost ist nach 4–6 Monaten wichtig für Stillkinder. Geburtsh Frauenheilk. 2011;71(07):615–16. DOI: 10.1055/s-0031-1280139.

214. Brown A, Jones SW, Rowan H. Baby-Led Weaning: The Evidence to Date. Current nutrition reports. 2017;6(2):148–56. DOI: 10.1007/s13668-017-0201-2.

215. Harrison M, Brodribb W, Hepworth J. A qualitative systematic review of maternal infant feeding practices in transitioning from milk feeds to family foods. Maternal & child nutrition. 2017;13(2). DOI: 10.1111/mcn.12360.

216. Wilken M. Das Essverhalten frühgeborener Säuglinge. Hebamme. 2016;29(05):290–94. DOI: 10.1055/s-0042-113353.

217. Buehler D, Castelletti S. Fütter-und Essstörungen bei Säuglingen und Kindern - Ein interdisziplinärer Ansatz. 2021.

218. Kwon KM, Shim JE, Kang M, Paik H-Y. Association between Picky Eating Behaviors and Nutritional Status in Early Childhood: Performance of a Picky Eating Behavior Questionnaire. Nutrients. 2017;9(5). DOI: 10.3390/nu9050463.

219. Wright CM, Parkinson KN, Shipton D, Drewett RF. How do toddler eating problems relate to their eating behavior, food preferences, and growth? Pediatrics. 2007;120(4):e1069-75. DOI: 10.1542/peds.2006-2961.

220. Katz RM, Hyche JK, Wingert EK. Pediatric Feeding Disorders: Feeding Children Who Can't or Won't Eat. In: Encyclopedia of Human Nutrition: Elsevier; 2013. p. 21–27.

221. Lai BP, Tang CS, Tse WK. A longitudinal study investigating disordered eating during the transition to motherhood among Chinese women in Hong Kong. The International journal of eating disorders. 2006;39(4):303–11. DOI: 10.1002/EAT.20266.

222. Birch L, Savage JS, Ventura A. Influences on the Development of Children's Eating Behaviours: From Infancy to Adolescence. Canadian journal of dietetic practice and research : a publication of Dietitians of Canada = Revue canadienne de la pratique et de la recherche en dietetique : une publication des Dietetistes du Canada. 2007;68(1):s1-s56.

223. Haines J, Haycraft E, Lytle L, Nicklaus S, Kok FJ, Merdji M, et al. Nurturing Children's Healthy Eating: Position statement. Appetite. 2019;137:124–33. DOI: 10.1016/j.appet.2019.02.007.

224. Gahagan S. Development of eating behavior: biology and context. Journal of developmental and behavioral pediatrics : JDBP. 2012;33(3):261–71. DOI: 10.1097/DBP.0b013e31824a7baa.

225. Scaglioni S, Arrizza C, Vecchi F, Tedeschi S. Determinants of children's eating behavior. The American journal of clinical nutrition. 2011;94(6 Suppl):2006S-2011S. DOI: 10.3945/ajcn.110.001685.

226. Scaglioni S, Cosmi V de, Ciappolino V, Parazzini F, Brambilla P, Agostoni C. Factors Influencing Children's Eating Behaviours. Nutrients. 2018;10(6). DOI: 10.3390/nu10060706.

227. Patrick H, Nicklas TA. A review of family and social determinants of children's eating patterns and diet quality. Journal of the American College of Nutrition. 2005;24(2):83–92. DOI: 10.1080/07315724.2005.10719448.

228. Roberts M, Pettigrew S. Psychosocial Influences on Children's Food Consumption. Psychology and Marketing. 2013;30(2):103–20. DOI: 10.1002/MAR.20591.

229. Zarnowiecki DM, Dollman J, Parletta N. Associations between predictors of children's dietary intake and socioeconomic position: a systematic review of the literature. Obesity reviews : an official journal of the International Association for the Study of Obesity. 2014;15(5):375–91. DOI: 10.1111/obr.12139.

230. Tan F, Zhao X, Yi R, Xu N, Zhang J. Analysis of Influence of Family Status on Dietary Behavior of Preschool Children through Data Samples: A Case Study of Eating Frequency of Western Fast Food. J. Phys.: Conf. Ser. 2020;1437(1):12117. DOI: 10.1088/1742-6596/1437/1/012117.

231. Campbell K, Crawford D, Jackson M, Cashel K, Worsley A, Gibbons K, et al. Family food environments of 5-6-year-old-children: does socioeconomic status make a difference? Asia Pacific journal of clinical nutrition. 2002;11 Suppl 3:S553-61. DOI: 10.1046/J.0964-7058.2002.00346.X.

232. Position of the American Dietetic Association: individual-, family-, school-, and community-based interventions for pediatric overweight. Journal of the American Dietetic Association. 2006;106(6):925–45. DOI: 10.1016/j.jada.2006.03.001.

233. Rodgers RF, Paxton SJ, Chabrol H. Effects of parental comments on body dissatisfaction and eating disturbance in young adults: a sociocultural model. Body image. 2009;6(3):171–77. DOI: 10.1016/j.bodyim.2009.04.004.

234. Hou F, Xu S, Zhao Y, Lu Q, Zhang S, Zu P, et al. Effects of emotional symptoms and life stress on eating behaviors among adolescents. Appetite. 2013;68:63–68. DOI: 10.1016/j.appet.2013.04.010.

235. Tate EB, Spruijt-Metz D, Pickering TA, Pentz MA. Two facets of stress and indirect effects on child diet through emotion-driven eating. Eating behaviors. 2015;18:84–90. DOI: 10.1016/j.eatbeh.2015.04.006.

236. Debeuf T, Verbeken S, van Beveren M-L, Michels N, Braet C. Stress and Eating Behavior: A Daily Diary Study in Youngsters. Frontiers in psychology. 2018;9:2657. DOI: 10.3389/fpsyg.2018.02657.

237. Rosen DS. Identification and management of eating disorders in children and adolescents. Pediatrics. 2010;126(6):1240–53. DOI: 10.1542/peds.2010-2821.

238. Lewinsohn PM, Holm-Denoma JM, Gau JM, Joiner TE, Striegel-Moore R, Bear P, et al. Problematic eating and feeding behaviors of 36-month-old children. The International journal of eating disorders. 2005;38(3):208–19. DOI: 10.1002/EAT.20175.

239. Hornberger LL, Lane MA. Identification and Management of Eating Disorders in Children and Adolescents. Pediatrics. 2021;147(1). DOI: 10.1542/peds.2020-040279.

240. Skemp-Arlt KM. Body Image Dissatisfaction and Eating Disturbances Among Children and Adolescents. Journal of Physical Education, Recreation & Dance. 2006;77(1):45–51. DOI: 10.1080/07303084.2006.10597813.

241. Golden NH, Katzman DK, Kreipe RE, Stevens SL, Sawyer SM, Rees J, et al. Eating disorders in adolescents. Journal of Adolescent Health. 2003;33(6):496–503. DOI: 10.1016/j.jadohealth.2003.08.004.

242. Hudson LD, Court AJ. What paediatricians should know about eating disorders in children and young people. Journal of paediatrics and child health. 2012;48(10):869–75. DOI: 10.1111/j.1440-1754.2012.02433.x.

243. Berksoy EA, Özyurt G, Anıl M, Üzüm Ö, Appak YÇ. ¿Puede un pediatra sospechar un trastorno de la conducta alimentaria? Un caso de comienzo precoz de anorexia nerviosa en un varón. Nutricion hospitalaria. 2018;35(2):499–502. DOI: 10.20960/nh.1744.

244. Drossman DA, Chang L, Deutsch JK, Ford AC, Halpert A, Kroenke K, et al. A Review of the Evidence and Recommendations on Communication Skills and the Patient-Provider Relationship: A Rome Foundation Working Team Report. Gastroenterology. 2021;161(5):1670-1688.e7. DOI: 10.1053/j.gastro.2021.07.037.

245. Crossing the Quality Chasm: A New Health System for the 21st Century. Washington (DC); 2001.

246. Davidson JE, Aslakson RA, Long AC, Puntillo KA, Kross EK, Hart J, et al. Guidelines for Family-Centered Care in the Neonatal, Pediatric, and Adult ICU. Critical care medicine. 2017;45(1):103–28. DOI: 10.1097/CCM.0000000000002169.

247. Franck LS, O'Brien K. The evolution of family-centered care: From supporting parent-delivered interventions to a model of family integrated care. Birth defects research. 2019;111(15):1044–59. DOI: 10.1002/bdr2.1521.

248. Park M, Giap T-T-T, Lee M, Jeong H, Jeong M, Go Y. Patient- and family-centered care interventions for improving the quality of health care: A review of systematic reviews. International journal of nursing studies. 2018;87:69–83. DOI: 10.1016/j.ijnurstu.2018.07.006.

249. Håkansson Eklund J, Holmström IK, Kumlin T, Kaminsky E, Skoglund K, Höglander J, et al. "Same same or different?" A review of reviews of person-centered and patient-centered care. Patient education and counseling. 2019;102(1):3–11. DOI: 10.1016/j.pec.2018.08.029.

250. Ko CJ, Kim R, Fortin AH, Spak JM, Hafler JP. Relationship-Centered Care in the Physician-Patient Interaction: Improving Your Understanding of Metacognitive Interventions. Cutis. 2021;107(6):320–24. DOI: 10.12788/cutis.0266.

251. Gómez-Cantarino S, García-Valdivieso I, Moncunill-Martínez E, Yáñez-Araque B, Ugarte Gurrutxaga MI. Developing a Family-Centered Care Model in the Neonatal Intensive Care Unit (NICU): A New Vision to Manage Healthcare. International journal of environmental research and public health. 2020;17(19). DOI: 10.3390/ijerph17197197.

252. Meert KL, Clark J, Eggly S. Family-centered care in the pediatric intensive care unit. Pediatric clinics of North America. 2013;60(3):761–72. DOI: 10.1016/j.pcl.2013.02.011.

253. Peterson E, Morgan R, Calhoun A. Improving Patient- and Family-Centered Communication in Pediatrics: A Review of Simulation-Based Learning. Pediatric annals. 2021;50(1):e32-e38. DOI: 10.3928/19382359-20201211-02.

254. Everhart JL, Haskell H, Khan A. Patient- and Family-Centered Care: Leveraging Best Practices to Improve the Care of Hospitalized Children. Pediatric clinics of North America. 2019;66(4):775–89. DOI: 10.1016/j.pcl.2019.03.005.

255. Popa-Velea O, Purcărea VL. Issues of therapeutic communication relevant for improving quality of care. Journal of medicine and life. 2014;7 Spec No. 4(Spec Iss 4):39–45.

256. Boquiren VM, Hack TF, Beaver K, Williamson S. What do measures of patient satisfaction with the doctor tell us? Patient education and counseling. 2015. DOI: 10.1016/j.pec.2015.05.020.

257. Georgopoulou S, Prothero L, D'Cruz DP. Physician-patient communication in rheumatology: a systematic review. Rheumatology international. 2018;38(5):763–75. DOI: 10.1007/s00296-018-4016-2.

258. Epstein RM, Street RL. PsycEXTRA Dataset; 2007.

259. Wild D, Nawaz H, Ullah S, Via C, Vance W, Petraro P. Teaching residents to put patients first: creation and evaluation of a comprehensive curriculum in patient-centered communication. BMC medical education. 2018;18(1):266. DOI: 10.1186/s12909-018-1371-3.

260. Carcone AI, Jacques-Tiura AJ, Brogan Hartlieb KE, Albrecht T, Martin T. Effective Patient-Provider Communication in Pediatric Obesity. Pediatric clinics of North America. 2016;63(3):525–38. DOI: 10.1016/j.pcl.2016.02.002.

261. Samdal GB, Eide GE, Barth T, Williams G, Meland E. Effective behaviour change techniques for physical activity and healthy eating in overweight and obese adults; systematic review and meta-regression analyses. The international journal of behavioral nutrition and physical activity. 2017;14(1):42. DOI: 10.1186/s12966-017-0494-y.

262. Arigliani M, Castriotta L, Pusiol A, Titolo A, Petoello E, Brun Peressut A, et al. Measuring empathy in pediatrics: validation of the Visual CARE measure. BMC pediatrics. 2018;18(1):57. DOI: 10.1186/s12887-018-1050-x.

263. Patel S, Pelletier-Bui A, Smith S, Roberts MB, Kilgannon H, Trzeciak S, et al. Curricula for empathy and compassion training in medical education: A systematic review. PloS one. 2019;14(8):e0221412. DOI: 10.1371/journal.pone.0221412.

264. Peterson EB, Boland KA, Bryant KA, McKinley TF, Porter MB, Potter KE, et al. Development of a Comprehensive Communication Skills Curriculum for Pediatrics Residents. Journal of graduate medical education. 2016;8(5):739–46. DOI: 10.4300/jgme-d-15-00485.1.

265. Zanon BP, Cremonese L, Ribeiro AC, Padoin SMdM, Paula CC de. Communication of bad news in pediatrics: integrative review. Revista brasileira de enfermagem. 2020;73 Suppl 4:e20190059. DOI: 10.1590/0034-7167-2019-0059.

266. Sinskey JL, Chang JM, Shibata GS, Infosino AJ, Rouine-Rapp K. Applying Conflict Management Strategies to the Pediatric Operating Room. Anesthesia and analgesia. 2019;129(4):1109–17. DOI: 10.1213/ANE.0000000000003991.

267. Schmidt A-K, Krones T. Aufklärungsgespräche in der Pädiatrie. Monatsschr Kinderheilkd. 2016;164(2):122–28. DOI: 10.1007/s00112-015-3483-8.

268. Collins K, Hopkins A, Shilkofski NA, Levine RB, Hernandez RG. Difficult Patient Encounters: Assessing Pediatric Residents' Communication Skills Training Needs. Cureus. 2018;10(9):e3340. DOI: 10.7759/cureus.3340.

269. Hilgenberg SL, Bogetz AL, Leibold C, Gaba D, Blankenburg RL. De-escalating Angry Caregivers: A Randomized Controlled Trial of a Novel Communication Curriculum for Pediatric Residents. Academic pediatrics. 2019;19(3):283–90. DOI: 10.1016/j.acap.2018.10.005.

270. Wilson A, Hurwitz CA, Smith M, Patino T, Kudalmana AS, Gallas M. Parents as Teachers: Teaching Pediatrics Residents the Art of Engaging in Difficult Conversations. Journal of graduate medical education. 2019;11(1):60–65. DOI: 10.4300/JGME-D-18-00180.1.

271. Cerda-Hegerl P. Interkulturelle Aspekte in der medizinischen Versorgung nicht-dokumentierter Migranten. Psychotherapie, Psychosomatik, medizinische Psychologie. 2008;58(3-4):136–45. DOI: 10.1055/s-2008-1067364.

272. Ullrich S, Briel D, Nesterko Y, Hiemisch A, Brähler E, Glaesmer H. Verständigung mit Patienten und Eltern mit Migrationshintergrund in der stationären allgemeinpädiatrischen Versorgung. Gesundheitswesen (Bundesverband der Arzte des Offentlichen Gesundheitsdienstes (Germany)). 2016;78(4):209–14. DOI: 10.1055/s-0042-102341.

273. Mărginean CO, Meliţ LE, Chinceşan M, Mureşan S, Georgescu AM, Suciu N, et al. Communication skills in pediatrics - the relationship between pediatrician and child. Medicine. 2017;96(43):e8399. DOI: 10.1097/MD.0000000000008399.

274. Bein T. Interkulturelle Kompetenz. Umgang mit Fremdheit in der Intensivmedizin. Der Anaesthesist. 2015;64(8):562–68. DOI: 10.1007/s00101-015-0069-8.

275. Strelow K-UR, Bahadır Ş, Stollhof B, Heeb RM, Buggenhagen H. Patient interviews in interprofessional and intercultural contexts (PinKo) - project report on

interdisciplinary competence development in students of medicine, pharmacy, and community interpreting. GMS journal for medical education. 2021;38(3):Doc67. DOI: 10.3205/zma001463.

276. Alizadeh S, Chavan M. Cultural competence dimensions and outcomes: a systematic review of the literature. Health & social care in the community. 2016;24(6):e117-e130. DOI: 10.1111/hsc.12293.

277. Rocque R, Leanza Y. A Systematic Review of Patients' Experiences in Communicating with Primary Care Physicians: Intercultural Encounters and a Balance between Vulnerability and Integrity. PloS one. 2015;10(10):e0139577. DOI: 10.1371/journal.pone.0139577.

278. Bell J, Condren M. Communication Strategies for Empowering and Protecting Children. The journal of pediatric pharmacology and therapeutics : JPPT : the official journal of PPAG. 2016;21(2):176–84. DOI: 10.5863/1551-6776-21.2.176.

279. Niinikoski H, Lagström H, Jokinen E, Siltala M, Rönnemaa T, Viikari J, et al. Impact of repeated dietary counseling between infancy and 14 years of age on dietary intakes and serum lipids and lipoproteins: the STRIP study. Circulation. 2007;116(9):1032–40. DOI: 10.1161/circulationaha.107.699447.

280. Niinikoski H, Pahkala K, Ala-Korpela M, Viikari J, Rönnemaa T, Lagström H, et al. Effect of repeated dietary counseling on serum lipoproteins from infancy to adulthood. Pediatrics. 2012;129(3):e704-13. DOI: 10.1542/peds.2011-1503.

281. Neville A, Jordan A, Beveridge JK, Pincus T, Noel M. Diagnostic Uncertainty in Youth With Chronic Pain and Their Parents. The journal of pain. 2019;20(9):1080–90. DOI: 10.1016/j.jpain.2019.03.004.

282. Hale AE, Smith AM, Christiana JS, Burch E, Schechter NL, Beinvogl BC, et al. Perceptions of Pain Treatment in Pediatric Patients With Functional Gastrointestinal Disorders. The Clinical journal of pain. 2020;36(7):550–57. DOI: 10.1097/ajp.0000000000000832.

283. Carter B. Chronic pain in childhood and the medical encounter: professional ventriloquism and hidden voices. Qualitative health research. 2002;12(1):28–41. DOI: 10.1177/104973230201200103.

284. Wangmo T, Clercq E de, Ruhe KM, Beck-Popovic M, Rischewski J, Angst R, et al. Better to know than to imagine: Including children in their health care. AJOB empirical bioethics. 2017;8(1):11–20. DOI: 10.1080/23294515.2016.1207724.

285. Breuner CC, Moreno MA. Approaches to the difficult patient/parent encounter. Pediatrics. 2011;127(1):163–69. DOI: 10.1542/peds.2010-0072.

286. Tomayko EJ, Tovar A, Fitzgerald N, Howe CL, Hingle MD, Murphy MP, et al. Parent Involvement in Diet or Physical Activity Interventions to Treat or Prevent Childhood Obesity: An Umbrella Review. Nutrients. 2021;13(9). DOI: 10.3390/nu13093227.

287. Resnicow K, McMaster F, Bocian A, Harris D, Zhou Y, Snetselaar L, et al. Motivational interviewing and dietary counseling for obesity in primary care: an RCT. Pediatrics. 2015;135(4):649–57. DOI: 10.1542/peds.2014-1880.

288. Ahmed U, Mahmood MS, Parsons M, O'callaghan H, Pawlik O, Chaudhary S, et al. A Systematic Review Looking at the Current Best Practices as well as Primary Care Practitioner's Views on the Diagnosis and Treatment of Childhood Obesity. Cureus. 2023;15(1):e34346. DOI: 10.7759/cureus.34346.

289. Enright G, Allman-Farinelli M, Redfern J. Effectiveness of Family-Based Behavior Change Interventions on Obesity-Related Behavior Change in Children: A Realist Synthesis. International journal of environmental research and public health. 2020;17(11). DOI: 10.3390/ijerph17114099.

290. Sisk BA, Friedrich AB, Mozersky J, Walsh H, DuBois J. Core Functions of Communication in Pediatric Medicine: an Exploratory Analysis of Parent and Patient Narratives. Journal of cancer education : the official journal of the American Association for Cancer Education. 2020;35(2):256–63. DOI: 10.1007/s13187-018-1458-x.

291. Sisk BA, Keenan M, Schulz GL, Kaye E, Baker JN, Mack JW, et al. Interdependent functions of communication with adolescents and young adults in oncology. Pediatric blood & cancer. 2022;69(4):e29588. DOI: 10.1002/pbc.29588.

292. Sisk BA, Zavadil JA, Blazin LJ, Baker JN, Mack JW, DuBois JM. Assume It Will Break: Parental Perspectives on Negative Communication Experiences in Pediatric Oncology. JCO oncology practice. 2021;17(6):e859-e871. DOI: 10.1200/op.20.01038.

293. Koksal I, Acikgoz A, Cakirli M. The Effect of a Father's Support on Breastfeeding: A Systematic Review. Breastfeeding medicine : the official journal of the Academy of Breastfeeding Medicine. 2022;17(9):711–22. DOI: 10.1089/bfm.2022.0058.

294. Ngoenthong P, Sansiriphun N, Fongkaew W, Chaloumsuk N. Integrative Review of Fathers' Perspectives on Breastfeeding Support. Journal of obstetric, gynecologic, and neonatal nursing : JOGNN. 2020;49(1):16–26. DOI: 10.1016/j.jogn.2019.09.005.

295. Abbass-Dick J, Brown HK, Jackson KT, Rempel L, Dennis C-L. Perinatal breastfeeding interventions including fathers/partners: A systematic review of the literature. Midwifery. 2019;75:41–51. DOI: 10.1016/j.midw.2019.04.001.

296. Mitchell-Box KM, Braun KL. Impact of male-partner-focused interventions on breastfeeding initiation, exclusivity, and continuation. Journal of human lactation : official journal of International Lactation Consultant Association. 2013;29(4):473–79. DOI: 10.1177/0890334413491833.

297. Mahesh PKB, Gunathunga MW, Arnold SM, Jayasinghe C, Pathirana S, Makarim MF, et al. Effectiveness of targeting fathers for breastfeeding promotion: systematic review and meta-analysis. BMC public health. 2018;18(1):1140. DOI: 10.1186/s12889-018-6037-x.

298. Tokhi M, Comrie-Thomson L, Davis J, Portela A, Chersich M, Luchters S. Involving men to improve maternal and newborn health: A systematic review of the effectiveness of interventions. PloS one. 2018;13(1):e0191620. DOI: 10.1371/journal.pone.0191620.

299. Stark LJ, Powers SW. Behavioral aspects of nutrition in children with cystic fibrosis. Current opinion in pulmonary medicine. 2005;11(6):539–42. DOI: 10.1097/01.mcp.0000183051.18611.e4.

300. Gonçalves JdA, Moreira EAM, Trindade EBSdM, Fiates GMR. Eating disorders in childhood and adolescence. Revista paulista de pediatria : orgao oficial da Sociedade de Pediatria de Sao Paulo. 2013;31(1):96–103. DOI: 10.1590/s0103-05822013000100016.

301. Hendy HM, Williams KE, Riegel K, Paul C. Parent mealtime actions that mediate associations between children's fussy-eating and their weight and diet. Appetite. 2010;54(1):191–95. DOI: 10.1016/j.appet.2009.10.006.

302. Tanaka A, Kawamura M, Yamada K, Morioka I. Association between teaching and support skills and subjective effectiveness of nutritional guidance of registered dietitians at hospitals in a Japanese prefecture. Environmental health and preventive medicine. 2014;19(1):72–80. DOI: 10.1007/s12199-013-0358-2.

303. Lok KYW, Chan RSM, Sea MMM, Woo J. Nutritionist's variation in counseling style and the effect on weight change of patients attending a community based lifestyle modification program. International journal of environmental research and public health. 2010;7(2):413–26. DOI: 10.3390/ijerph7020413.

304. Verheijden MW, van der Veen JE, Bakx JC, Akkermans RP, van den Hoogen HJM, van Staveren WA, et al. Stage-matched nutrition guidance: stages of change and fat consumption in Dutch patients at elevated cardiovascular risk. Journal of nutrition education and behavior. 2004;36(5):228–37. DOI: 10.1016/s1499-4046(06)60385-0.

305. Brug J, Spikmans F, Aartsen C, Breedveld B, Bes R, Fereira I. Training dietitians in basic motivational interviewing skills results in changes in their counseling style and in lower saturated fat intakes in their patients. Journal of nutrition education and behavior. 2007;39(1):8–12. DOI: 10.1016/j.jneb.2006.08.010.

306. Britton B, Baker AL, Wolfenden L, Wratten C, Bauer J, Beck AK, et al. Eating As Treatment (EAT): A Stepped-Wedge, Randomized Controlled Trial of a Health Behavior Change Intervention Provided by Dietitians to Improve Nutrition in Patients With Head and Neck Cancer Undergoing Radiation Therapy (TROG 12.03). International journal of radiation oncology, biology, physics. 2019;103(2):353–62. DOI: 10.1016/j.ijrobp.2018.09.027.

307. Adam LM, Jarman M, Barker M, Manca DP, Lawrence W, Bell RC. Use of healthy conversation skills to promote healthy diets, physical activity and gestational weight gain: Results from a pilot randomised controlled trial. Patient education and counseling. 2020;103(6):1134–42. DOI: 10.1016/j.pec.2020.01.001.

308. Vincze L, Rollo ME, Hutchesson MJ, Callister R, Collins CE. VITAL change for mums: a feasibility study investigating tailored nutrition and exercise care delivered by video-consultations for women 3-12 months postpartum. Journal of human nutrition and dietetics : the official journal of the British Dietetic Association. 2018;31(3):337–48. DOI: 10.1111/jhn.12549.

309. Resnicow K, Harris D, Wasserman R, Schwartz RP, Perez-Rosas V, Mihalcea R, et al. Advances in Motivational Interviewing for Pediatric Obesity: Results of the Brief Motivational Interviewing to Reduce Body Mass Index Trial and Future Directions. Pediatric clinics of North America. 2016;63(3):539–62. DOI: 10.1016/j.pcl.2016.02.008.

310. Pollak KI, Nagy P, Bigger J, Bilheimer A, Lyna P, Gao X, et al. Effect of teaching motivational interviewing via communication coaching on clinician and patient satisfaction in primary care and pediatric obesity-focused offices. Patient education and counseling. 2016;99(2):300–03. DOI: 10.1016/j.pec.2015.08.013.

311. Laroche HH, Park-Mroch J, O'Shea A, Rice S, Cintron Y, Engebretsen B. Resource mobilization combined with motivational interviewing to promote healthy behaviors and healthy weight in low-income families: An intervention feasibility study. SAGE open medicine. 2022;10:20503121221102706. DOI: 10.1177/20503121221102706.

312. Braun A, Portner J, Xu M, Weaver L, Pratt K, Darragh A, et al. Preliminary Support for the Use of Motivational Interviewing to Improve Parent/Adult Caregiver Behavior for Obesity and Cancer Prevention. International journal of environmental research and public health. 2023;20(6). DOI: 10.3390/ijerph20064726.

313. Woolford SJ, Resnicow K, Davis MM, Nichols LP, Wasserman RC, Harris D, et al. Cost-effectiveness of a motivational interviewing obesity intervention versus usual care in pediatric primary care offices. Obesity (Silver Spring, Md.). 2022;30(11):2265–74. DOI: 10.1002/oby.23560.

314. Mutschler C, Naccarato E, Rouse J, Davey C, McShane K. Realist-informed review of motivational interviewing for adolescent health behaviors. Systematic reviews. 2018;7(1):109. DOI: 10.1186/s13643-018-0767-9.

315. Taveras EM, Marshall R, Kleinman KP, Gillman MW, Hacker K, Horan CM, et al. Comparative effectiveness of childhood obesity interventions in pediatric primary care: a cluster-randomized clinical trial. JAMA pediatrics. 2015;169(6):535–42. DOI: 10.1001/jamapediatrics.2015.0182.

316. Kłósek P, Grosicki S, Całyniuk B. Improving the effectiveness of obesity treatment by combining a diet and motivational techniques. Roczniki Panstwowego Zakladu Higieny. 2018;69(3):299–305.

317. Walker MH, Murimi MW, Kim Y, Hunt A, Erickson D, Strimbu B. Multiple point-of-testing nutrition counseling sessions reduce risk factors for chronic disease among older adults. Journal of nutrition in gerontology and geriatrics. 2012;31(2):146–57. DOI: 10.1080/21551197.2012.678233.

318. Digenio AG, Mancuso JP, Gerber RA, Dvorak RV. Comparison of methods for delivering a lifestyle modification program for obese patients: a randomized trial. Annals of internal medicine. 2009;150(4):255-62. DOI: 10.7326/0003-4819-150-4-200902170-00006.

319. Else V, Chen Q, Cortez AB, Koebnick C. Sustainability of weight loss from a family-centered pediatric weight management program integrated in primary care. BMC health services research. 2022;22(1):12. DOI: 10.1186/s12913-021-07361-9.

320. Hassapidou M, Duncanson K, Shrewsbury V, Ells L, Mulrooney H, Androutsos O, et al. EASO and EFAD Position Statement on Medical Nutrition Therapy for the Management of Overweight and Obesity in Children and Adolescents. Obesity facts. 2023;16(1):29-52. DOI: 10.1159/000527540.

321. Michie S, Abraham C, Whittington C, McAteer J, Gupta S. Effective techniques in healthy eating and physical activity interventions: a meta-regression. Health psychology : official journal of the Division of Health Psychology, American Psychological Association. 2009;28(6):690-701. DOI: 10.1037/a0016136.

322. Lara J, Evans EH, O'Brien N, Moynihan PJ, Meyer TD, Adamson AJ, et al. Association of behaviour change techniques with effectiveness of dietary interventions among adults of retirement age: a systematic review and meta-analysis of randomised controlled trials. BMC medicine. 2014;12:177. DOI: 10.1186/s12916-014-0177-3.

323. Paine K, Parker S, Denney-Wilson E, Lloyd J, Randall S, McNamara C, et al. In it for the long haul: the complexities of managing overweight in family practice: qualitative thematic analysis from the Health eLiteracy for Prevention in General Practice (HeLP-GP) trial. BMC primary care. 2023;24(1):57. DOI: 10.1186/s12875-023-01995-w.

324. Dombrowski SU, Sniehotta FF, Avenell A, Johnston M, MacLennan G, Araújo-Soares V. Identifying active ingredients in complex behavioural interventions for obese adults with obesity-related co-morbidities or additional risk factors for co-morbidities: a systematic review. Health Psychology Review. 2012;6(1):7-32. DOI: 10.1080/17437199.2010.513298.

325. Nowicka P, Pietrobelli A, Flodmark C-E. Low-intensity family therapy intervention is useful in a clinical setting to treat obese and extremely obese children. International journal of pediatric obesity : IJPO : an official journal of the International Association for the Study of Obesity. 2007;2(4):211-17. DOI: 10.1080/17477160701379810.

326. Bridge GL, Willis TA, Evans CEL, Roberts KPJ, Rudolf M. The impact of HENRY on parenting and family lifestyle: Exploratory analysis of the mechanisms for change. Child: care, health and development. 2019;45(6):850-60. DOI: 10.1111/cch.12694.

327. Akgul Gundogdu N, Sevig EU, Guler N. The effect of the solution-focused approach on nutrition-exercise attitudes and behaviours of overweight and obese

adolescents: Randomised controlled trial. Journal of clinical nursing. 2018;27(7-8):e1660-e1672. DOI: 10.1111/jocn.14246.

328. Marcano-Olivier M, Pearson R, Ruparell A, Horne PJ, Viktor S, Erjavec M. A low-cost Behavioural Nudge and choice architecture intervention targeting school lunches increases children's consumption of fruit: a cluster randomised trial. The international journal of behavioral nutrition and physical activity. 2019;16(1):20. DOI: 10.1186/s12966-019-0773-x.

329. Sharps MA, Thomas E, Blissett JM. Using pictorial nudges of fruit and vegetables on tableware to increase children's fruit and vegetable consumption. Appetite. 2020;144:104457. DOI: 10.1016/j.appet.2019.104457.

330. Jebeile H, Gow ML, Baur LA, Garnett SP, Paxton SJ, Lister NB. Treatment of obesity, with a dietary component, and eating disorder risk in children and adolescents: A systematic review with meta-analysis. Obesity reviews : an official journal of the International Association for the Study of Obesity. 2019;20(9):1287–98. DOI: 10.1111/obr.12866.

331. Jebeile H, Lister NB, Baur LA, Garnett SP, Paxton SJ. Eating disorder risk in adolescents with obesity. Obesity reviews : an official journal of the International Association for the Study of Obesity. 2021;22(5):e13173. DOI: 10.1111/obr.13173.

332. Stabouli S, Erdine S, Suurorg L, Jankauskienė A, Lurbe E. Obesity and Eating Disorders in Children and Adolescents: The Bidirectional Link. Nutrients. 2021;13(12). DOI: 10.3390/nu13124321.

333. Lister NB, Baur LA, Paxton SJ, Jebeile H. Contextualising Eating Disorder Concerns for Paediatric Obesity Treatment. Current obesity reports. 2021;10(3):322–31. DOI: 10.1007/s13679-021-00440-2.

334. Lister NB, Baur LA, Paxton SJ, Garnett SP, Ahern AL, Wilfley DE, et al. Eating Disorders In weight-related Therapy (EDIT) Collaboration: rationale and study design. Nutrition research reviews. 2024;37(1):32–42. DOI: 10.1017/s0954422423000045.

335. Keski-Rahkonen A. Epidemiology of binge eating disorder: prevalence, course, comorbidity, and risk factors. Current opinion in psychiatry. 2021;34(6):525–31. DOI: 10.1097/yco.0000000000000750.

336. Agüera Z, Lozano-Madrid M, Mallorquí-Bagué N, Jiménez-Murcia S, Menchón JM, Fernández-Aranda F. Übersicht zu Binge-eating und Adipositas. Neuropsychiatrie : Klinik, Diagnostik, Therapie und Rehabilitation : Organ der Gesellschaft Osterreichischer Nervenarzte und Psychiater. 2021;35(2):57–67. DOI: 10.1007/s40211-020-00346-w.

337. Brown TA, Forney KJ, Klein KM, Grillot C, Keel PK. A 30-year longitudinal study of body weight, dieting, and eating pathology across women and men from late adolescence to later midlife. Journal of abnormal psychology. 2020;129(4):376–86. DOI: 10.1037/abn0000519.

338. Stice E, Johnson S, Turgon R. Eating Disorder Prevention. The Psychiatric clinics of North America. 2019;42(2):309–18. DOI: 10.1016/j.psc.2019.01.012.

339. Culkin A, Gabe SM, Madden AM. Improving clinical outcome in patients with intestinal failure using individualised nutritional advice. Journal of human nutrition and dietetics : the official journal of the British Dietetic Association. 2009;22(4):290-8; quiz 300-1. DOI: 10.1111/j.1365-277x.2009.00954.x.

340. Mustila T, Raitanen J, Keskinen P, Saari A, Luoto R. Pragmatic controlled trial to prevent childhood obesity in maternity and child health care clinics: pregnancy and infant weight outcomes (the VACOPP Study). BMC pediatrics. 2013;13:80. DOI: 10.1186/1471-2431-13-80.

341. Enwald HPK, Huotari M-LA. Preventing the obesity epidemic by second generation tailored health communication: an interdisciplinary review. Journal of medical Internet research. 2010;12(2):e24. DOI: 10.2196/jmir.1409.

342. Kracht CL, Hutchesson M, Ahmed M, Müller AM, Ashton LM, Brown HM, et al. E-&mHealth interventions targeting nutrition, physical activity, sedentary behavior, and/or obesity among children: A scoping review of systematic reviews and meta-analyses. Obesity reviews : an official journal of the International Association for the Study of Obesity. 2021;22(12):e13331. DOI: 10.1111/obr.13331.

343. Fiedler J, Eckert T, Wunsch K, Woll A. Key facets to build up eHealth and mHealth interventions to enhance physical activity, sedentary behavior and nutrition in healthy subjects - an umbrella review. BMC public health. 2020;20(1):1605. DOI: 10.1186/s12889-020-09700-7.

344. Tully L, Burls A, Sorensen J, El-Moslemany R, O'Malley G. Mobile Health for Pediatric Weight Management: Systematic Scoping Review. JMIR mHealth and uHealth. 2020;8(6):e16214. DOI: 10.2196/16214.

345. Bert F, Giacometti M, Gualano MR, Siliquini R. Smartphones and health promotion: a review of the evidence. Journal of medical systems. 2014;38(1):9995. DOI: 10.1007/s10916-013-9995-7.

346. Holmberg C, Berg C, Dahlgren J, Lissner L, Chaplin JE. Health literacy in a complex digital media landscape: Pediatric obesity patients' experiences with online weight, food, and health information. Health informatics journal. 2019;25(4):1343–57. DOI: 10.1177/1460458218759699.

347. Chew CSE, Davis C, Lim JKE, Lim CMM, Tan YZH, Oh JY, et al. Use of a Mobile Lifestyle Intervention App as an Early Intervention for Adolescents With Obesity: Single-Cohort Study. Journal of medical Internet research. 2021;23(9):e20520. DOI: 10.2196/20520.

348. Vaillancourt H, Légaré F, Gagnon M-P, Lapointe A, Deschênes S-M, Desroches S. Exploration of shared decision-making processes among dieticians and patients during a consultation for the nutritional treatment of dyslipidaemia. Health expectations : an international journal of public participation in health care and health policy. 2015;18(6):2764–75. DOI: 10.1111/hex.12250.

349. Deschênes S-M, Gagnon M-P, Légaré F, Lapointe A, Turcotte S, Desroches S. Psychosocial factors of dietitians' intentions to adopt shared decision making behaviours: a cross-sectional survey. PloS one. 2013;8(5):e64523. DOI: 10.1371/journal.pone.0064523.

350. Szucs KA, Miracle DJ, Rosenman MB. Breastfeeding knowledge, attitudes, and practices among providers in a medical home. Breastfeeding medicine : the official journal of the Academy of Breastfeeding Medicine. 2009;4(1):31–42. DOI: 10.1089/bfm.2008.0108.

351. Lowenstein LM, Perrin EM, Campbell MK, Tate DF, Cai J, Ammerman AS. Primary care providers' self-efficacy and outcome expectations for childhood obesity counseling. Childhood obesity (Print). 2013;9(3):208–15. DOI: 10.1089/chi.2012.0119.

352. Wynn K, Trudeau JD, Taunton K, Gowans M, Scott I. Nutrition in primary care: current practices, attitudes, and barriers. Canadian family physician Medecin de famille canadien. 2010;56(3):e109-16.

353. Ozer EM, Adams SH, Lustig JL, Gee S, Garber AK, Gardner LR, et al. Increasing the screening and counseling of adolescents for risky health behaviors: a primary care intervention. Pediatrics. 2005;115(4):960–68. DOI: 10.1542/peds.2004-0520.

354. Buckelew SM, Adams SH, Irwin CE, Gee S, Ozer EM. Increasing clinician self-efficacy for screening and counseling adolescents for risky health behaviors: results of an intervention. The Journal of adolescent health : official publication of the Society for Adolescent Medicine. 2008;43(2):198–200. DOI: 10.1016/j.jadohealth.2008.01.018.

355. Gonzalez JL, Gilmer L. Obesity prevention in pediatrics: A pilot pediatric resident curriculum intervention on nutrition and obesity education and counseling. Journal of the National Medical Association. 2006;98(9):1483–88.

356. Dumic A, Miskulin I, Matic Licanin M, Mujkic A, Cacic Kenjeric D, Miskulin M. Nutrition Counselling Practices among General Practitioners in Croatia. International journal of environmental research and public health. 2017;14(12). DOI: 10.3390/ijerph14121499.

357. Perrin EM, Flower KB, Garrett J, Ammerman AS. Preventing and treating obesity: pediatricians' self-efficacy, barriers, resources, and advocacy. Ambulatory pediatrics : the official journal of the Ambulatory Pediatric Association. 2005;5(3):150–56. DOI: 10.1367/a04-104r.1.

358. Adams KM, Lindell KC, Kohlmeier M, Zeisel SH. Status of nutrition education in medical schools. The American journal of clinical nutrition. 2006;83(4):941S-944S. DOI: 10.1093/ajcn/83.4.941s.

359. Adams KM, Kohlmeier M, Zeisel SH. Nutrition education in U.S. medical schools: latest update of a national survey. Academic medicine : journal of the Association of American Medical Colleges. 2010;85(9):1537–42. DOI: 10.1097/acm.0b013e3181eab71b.

360. Crowley J, Ball L, Hiddink GJ. Nutrition in medical education: a systematic review. The Lancet. Planetary health. 2019;3(9):e379-e389. DOI: 10.1016/s2542-5196(19)30171-8.

361. Chung M, van Buul VJ, Wilms E, Nellessen N, Brouns FJPH. Nutrition education in European medical schools: results of an international survey. European journal of clinical nutrition. 2014;68(7):844–46. DOI: 10.1038/ejcn.2014.75.

362. Cuerda C, Muscaritoli M, Donini LM, Baqué P, Barazzoni R, Gaudio E, et al. Nutrition education in medical schools (NEMS). An ESPEN position paper. Clinical nutrition (Edinburgh, Scotland). 2019;38(3):969–74. DOI: 10.1016/j.clnu.2019.02.001.
